# Hot Carrier Trapping and It's Influence to the Carrier Diffusion in CsPbBr_3_ Perovskite Film Revealed by Transient Absorption Microscopy

**DOI:** 10.1002/advs.202403507

**Published:** 2024-05-10

**Authors:** Jianchang Lv, Ao Liu, Danli Shi, Minjie Li, Xi Liu, Yan Wan

**Affiliations:** ^1^ College of Chemistry Beijing Normal University Beijing 100875 P. R. China

**Keywords:** carrier diffusion, hot carrier, imaging, perovskite

## Abstract

The defects in perovskite film can cause charge carrier trapping which shortens carrier lifetime and diffusion length. So defects passivation has become promising for the perovskite studies. However, how defects disturb the carrier transport and how the passivating affects the carrier transport in CsPbBr_3_ are still unclear. Here the carrier dynamics and diffusion processes of CsPbBr_3_ and LiBr passivated CsPbBr_3_ films are investigated by using transient absorption spectroscopy and transient absorption microscopy. It's found that there is a fast hot carrier trapping process with the above bandgap excitation, and the hot carrier trapping would decrease the population of cold carriers which are diffusible, then lower the carrier diffusion constant. It's proved that LiBr can passivate the defect and lower the trapping probability of hot carriers, thus improve the carrier diffusion rate. The finding demonstrates the influence of hot carrier trapping to the carrier diffusion in CsPbBr_3_ film.

## Graphical abstract

The hot carrier trapping will decrease the population of cold carriers which are diffusible, and then lower the carrier diffusion constant in CsPbBr_3_ film. It's proved that LiBr can passivate the defect and lower the trapping probability of hot carriers, thus improve the carrier diffusion rate. It demonstrates the influence of hot carrier trapping to the carrier diffusion in CsPbBr_3_ perovskite.

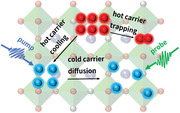

## 1 Introduction

All‐inorganic lead halide perovskite as an emerging material has been successfully applied on the solar cell, light‐emitting diodes (LEDs), and photodetectors.^[^ ^1^ ^]^ For example, the authentication efficiency of CsPbBr_3_ perovskite solar cell has reached 11.08%, and the external quantum efficiency of all‐inorganic perovskite green LEDs has surpassed 16%, in the past few years.^[^ ^2^ ^]^ However, it is well known that solution‐processed perovskite film would generate many defects. The defects could cause charge carrier trapping which shortens carrier lifetime and diffusion length. The carrier lifetime and diffusion length are critical factors that determining the performance of perovskite devices.^[^ ^3^ ^]^ So defects passivation has become a promising direction for the perovskite studies.^[^ ^4^ ^]^

The diffusion constant (*D*) of carrier describes the rate of carrier diffusing from high concentration to low concentration. It determines the carrier diffusion length with carrier lifetime *τ* (diffusion length = $\sqrt{2D\tau }$). Guo et al. reported the long‐range carrier transport (≈220 nm in 2 ns) in CH_3_NH_3_PbI_3_ perovskite films by using transient absorption microscopy (TAM).^[^ ^5^ ^]^ Wang et al. reported that doping Rb, Cs, and K cation into perovskite thin films could increase hot carrier migration by using ultrafast transient absorption spectroscopy (TA) and TAM.^[^ ^6^ ^]^ The surface functionalization of MAPbI_3_ perovskite with phenethylammonium iodide, can enhance carrier diffusion constant in the near‐surface regions from 0.6 to 1 cm^2^ s^−1^.^[^ ^7^ ^]^ Recently, Wu et al. reported that LiBr could effectively passivate defects in CsPbBr_3_ perovskite thin films and enhance the external quantum efficiency and stability of LEDs.^[^ ^8^ ^]^ However, how do defects disturb the carrier transport and how do the passivating affect the carrier transport in CsPbBr_3_ perovskite are still unclear.

Here we have investigated the carrier dynamics and carrier diffusion process of CsPbBr_3_ and LiBr passivated CsPbBr_3_ perovskite films by using TA and TAM. The TA results show that there is a fast hot carrier trapping process with the above bandgap excitation, but the cold carrier trapping is not obviously with near band edge excitation. The TAM measurements indicate that the diffusion constant enlarges with the increasing of initial cold carrier density. And the hot carrier trapping would decrease the population of cold carriers which are diffusible, then lower the carrier diffusion constant. But LiBr can passivate the defect and reduce the trapping probability of hot carrier, thus improve the carrier diffusion rate.

## 2 Results and Discussion

### 2.1 Steady‐State Spectra and Characterizations

We have synthesized two polycrystalline perovskite films following the method previously reported by Wu et al (see Experimental Section for detail).^[^ ^8^ ^]^ The perovskite film was spin‐coated on the glass substrate directly. The pristine perovskite film is denoted as CsPbBr_3_ and the perovskite film with LiBr added is denoted as LiBr‐CsPbBr_3_. LiBr can efficiently passivate the defects generated by Br^−^ vacancies and reduce trap state density.^[^ ^8^ ^]^

**Figure 1:**
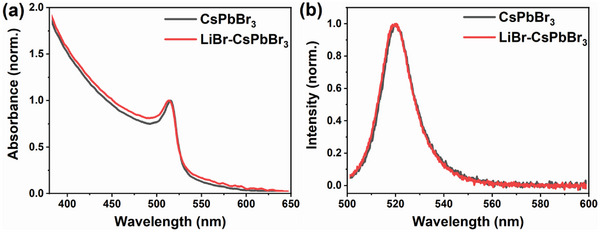
a) The normalized absorption spectra of CsPbBr_3_ and LiBr‐CsPbBr_3_ films. b) The normalized PL spectra of CsPbBr_3_ and LiBr‐CsPbBr_3_ films excited at ≈2.74 eV (453 nm).

**Figure** **1** shows the absorption and photoluminescence (PL) spectra for CsPbBr_3_ and LiBr‐CsPbBr_3_. The absorption spectra of both samples are similar and the bandgap of them is almost the same (2.353 eV for CsPbBr_3_ and 2.355 eV for LiBr‐CsPbBr_3_, see Figure S1, Supporting Information). Both of the PL spectra show a peak with the center wavelength at ≈520 nm. If Li^+^ entered the lattice, the lattice would be distorted due to the different atomic sizes with Cs^+^, causing changes in the energy levels, thus changing the peak position of the steady‐state spectra.^[^ ^9^ ^]^ However, the steady‐state spectra are not shifting with the addition of LiBr, so Li^+^ should not enter the CsPbBr_3_ lattice, which is consistent with that in a previous report.^[^ ^8^ ^]^

As shown in the PL microscopy and scanning electron microscopy (SEM) images (Figures S2 and S3, Supporting Information), there are many voids between grains in the pristine CsPbBr_3_ film obviously, but the LiBr‐CsPbBr_3_ film is much flatter. The TAM synchronous scanning image is shown in Figure S4 (Supporting Information). In this measurement, pump and probe beams overlap in space and scan synchronously (see Experimental Section for detail). So it reflects the morphology of perovskite films.^[^ ^5^ ^]^ Summing up the above, it's proved that the LiBr passivating can reduce voids and grain boundaries in CsPbBr_3_ film.

In addition, the cross‐section of perovskite films was measured by SEM to confirm the thickness of the film. As shown in Figure S5 (Supporting Information), the thickness is ≈300 and ≈240 nm for CsPbBr_3_ and LiBr‐CsPbBr_3_, respectively. Figure S6 (Supporting Information) displays the X‐ray diffraction (XRD) spectra of CsPbBr_3_ and LiBr‐CsPbBr_3_. The XRD pattern of both films contains three main peaks at 15.2^°^, 21.6^°,^ and 30.7^°^, which are attributed to the (100), (110), and (200) faces, respectively.^[^ ^8^, ^10^ ^]^

### 2.2 Femtosecond Transient Absorption Spectroscopy and Carrier Dynamics

**Figure 2:**
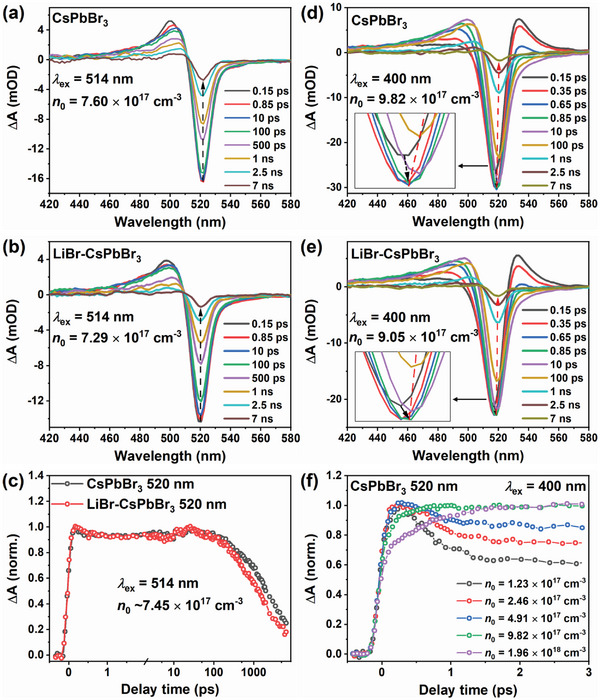
The TA spectra of a) CsPbBr_3_ and b) LiBr‐CsPbBr_3_ films at several delay times under 514 nm excitation with the initial carrier density (*n*_0_) ≈7.45×10^17^ cm^−3^. The dashed arrow indicates the change of the PB peak signal. c) Normalized kinetic profiles probed at 520 nm for CsPbBr_3_ and LiBr‐CsPbBr_3_ films under 514 nm excitation. The TA spectra of d) CsPbBr_3_ and e) LiBr‐CsPbBr_3_ films at several delay times under 400 nm excitation with the initial carrier density (*n*_0_) ≈9.44×10^17^ cm^−3^. f) Normalized kinetic profiles probed at 520 nm in the early time with different initial carrier density for CsPbBr_3_ film under 400 nm excitation.

**Figure** **2**a,b shows TA spectra of CsPbBr_3_ and LiBr‐CsPbBr_3_ under near band edge excitation (514 nm, 2.4 eV). The calculation of initial carrier density (*n*_0_) is described in Supplementary Note in the Supporting Information. The TA spectra of both films show two spectral shapes, a negative photoinduced bleaching (PB) peak at ≈520 nm and a positive photoinduced absorption (PA) peak at ≈500 nm. The PA band with energy above the bandgap is assigned to the absorption of the photoinduced carriers.^[^ ^11^ ^]^ The PB band is caused by the state filling of carriers at the band edge.^[^ ^12^ ^]^ Since the exciton binding energy in CsPbBr_3_ is very small (≈40 meV),^[^ ^13^ ^]^ excitons would dissociate into free carriers rapidly under our excitation density (*n*_0_ = 10^17^−10^18^ cm^−3^). So the effect of excitons could be negligible (see Supplementary Note in Supporting Information for detail). The PB signal reaches its maximum at 0.15 ps (IRF is ≈0.1 ps) and decays subsequently without peak shifting. As shown in Figure 2c, the PB decay of LiBr‐CsPbBr_3_ is faster than that of CsPbBr_3_.

We have also measured the TA spectra of two films under different initial carrier density (Figures S7 and S8, Supporting Information). The normalized PB dynamics at 520 nm are shown in Figure S9 (Supporting Information), and the carrier decays faster with the pump fluence increasing. It indicates that there is a high‐order carrier recombination process.^[^ ^12^, ^14^ ^]^ Figure S10 (Supporting Information) shows the linear fitting to the bimolecular recombination rate equation, the specific calculation details can be found in our previous publication.^[^ ^14^ ^]^ The good linearity of the reciprocal of kinetic traces indicates that the bleach recovery is primarily carried out through bimolecular recombination.^[^ ^12^, ^14^ ^]^ The calculated bimolecular recombination rate constants *B* are 1.1 × 10^−9^ and 1.8 × 10^−9^ cm^3^ s^−1^ for CsPbBr_3_ and LiBr‐CsPbBr_3_, respectively. The bimolecular recombination rate of LiBr‐CsPbBr_3_ is faster than that of CsPbBr_3_, which is consistent with Figure 2c. Note that the cold carrier (near band edge excited) trapping is not quite obviously under different initial carrier density, as shown by the PB dynamics in Figure S9 (Supporting Information). It implies that the trapped cold carriers could detrap easily.

We have measured the TA spectra under above bandgap excitation (400 nm, 3.1 eV) to examine the hot carrier dynamics. As shown in Figure 2d,e, the difference from 514 nm excitation is that a new PA peak (≈530 nm) appears, which is assigned to the bandgap renormalization of hot carriers.^[^ ^6^, ^15^ ^]^ The PB peaks at 518 nm initially, which is blue‐shifted comparing with the band edge excitation. It's ascribed to the Burstein−Moss band‐filling effect.^[^ ^12^ ^]^ The trade‐off from Burstein−Moss effect (blue‐shift) and bandgap renormalization (red‐shift) results in a slightly red‐shifting of PB peak within 1 ps (see the inset of Figure 2d,e).^[^ ^6^ ^]^

We have also measured the carrier‐density dependent TA spectra under 400 nm excitation for two samples (Figures S11 and S12, Supporting Information). Figure 2f shows the normalized PB kinetics of CsPbBr_3_ within 3 ps with different initial carrier density. Under lower *n*_0_, it appears a fast decay in the initial 2 ps. And with *n*_0_ increasing, the fast decay is disappeared, but is replaced by a rising process. The rising process is usually assigned to the hot carrier cooling, and it would slow down with the increasing of pump fluence due to the hot phonon bottleneck effect.^[^ ^16^ ^]^ The fast decay of the PB signal is interesting. Some reports consider it as the cold carrier trapping.^[^ ^17^ ^]^ But in this study, the fast decay process occurs only when the pump energy is above the bandgap as shown in Figure S13 (Supporting Information), so it should not be the trapping of the cold carrier. The initial carrier density in our TA experiment was much lower than 10^19^ cm^−3^, so the Auger recombination process can also be excluded. Hence, we think that the fast decay is related to hot carrier trapping. In other words, only the hot carrier could be trapped by the defect state. This perspective has been reported by previous studies.^[^ ^18^ ^]^ It's thought that the defect tolerance of material would decrease when the carrier obtained higher excess energies (higher carrier temperature), and then the trapping rate becomes faster.^[^ ^18a^ ^]^

### 2.3 Direct Imaging of Carrier Diffusion by Transient Absorption Microscopy

We have used TAM to directly image the carrier transport process in the two films. The pump position was fixed, while the probe beam was scanning relative to the pump beam by the Galvo mirror, and the pump‐induced change in probe transmission (Δ*T*) is plotted as a function of probe distance from the pump to form an image (for more details see Experimental Section).^[^ ^5^, ^19^ ^]^ We used the pump beam at 490 nm as the near band edge excitation and 400 nm as the above bandgap excitation. 520 nm was chosen for probing the PB signal.

The 2D TAM images are shown in Figure S14 (Supporting Information) for the 490 nm excitation and Figure S15 (Supporting Information) for the 400 nm excitation. At 0 ps, the TAM image represents the initial carrier population *n*(*x*, *y*, 0) created by the Gaussian pump beam with variance of σ(0)_*x*_ and σ(0)_*y*_ at the position (*x*_0_, *y*_0_):^[^ ^5^, ^19^, ^20^ ^]^

(1) $$n\;\left(x,y,0\right)=\;Nexp\left[-\frac{{\left(x-x_{0}\right)}^{2}}{2\sigma {\left(0\right)}_{x}^{2}}-\frac{{\left(y-y_{0}\right)}^{2}}{2\sigma {\left(0\right)}_{y}^{2}}\right]$$

At a later time *t*, the diffused carrier population also follows a Gaussian function. σ(*t*)_*x*_ and σ(*t*)_*y*_ are the variance of the Gaussian distribution along the x and y axes over time:

(2) $$n\;\left(x,y,t\right)=\;Nexp\left[-\frac{{\left(x-x_{0}\right)}^{2}}{2\sigma {\left(t\right)}_{x}^{2}}-\frac{{\left(y-y_{0}\right)}^{2}}{2\sigma {\left(t\right)}_{y}^{2}}\right]$$

As shown in Figures S14 and S15 (Supporting Information), the TAM images of two samples indicate the carriers have significantly diffused. Moreover, the diffusion constant could be extracted from the broadening of Gaussian distribution. The carrier transport of perovskite thin film is isotropic $(\sigma {\left(t\right)}^{2}\;=\;\sigma {\left(t\right)}_{x}^{2}\;=\;\sigma {\left(t\right)}_{y}^{2}\;)$,^[^ ^6^, ^15^ ^]^ therefore *L* ^2^ = σ(*t*)^2^  − σ(0)^2^ reflects the carrier diffusion distance *L* at the delay time *t*. The diffusion constant *D* is given by:^[^ ^6^, ^15^, ^20^, ^21^ ^]^

(3) $$D\;=\frac{\sigma {\left(t\right)}^{2}-\sigma {\left(0\right)}^{2}}{2t}\;=\frac{L^{2}}{2t}\;$$

We have extracted the diffusion constant by linear fitting of the time evolution of *L* ^2^ as shown in Figure S16 (Supporting Information). *D* is 1.2 cm^2^ s^−1^ for CsPbBr_3_ and 1.7 cm^2^ s^−1^ for LiBr‐CsPbBr_3_ under 490 nm excitation. It indicates that LiBr passivating has enhanced the diffusion rate of carriers. Under 400 nm excitation, *D* are 0.40 and 1.1 cm^2^ s^−1^ for CsPbBr_3_ and LiBr‐CsPbBr_3_, respectively.

**Figure 3:**
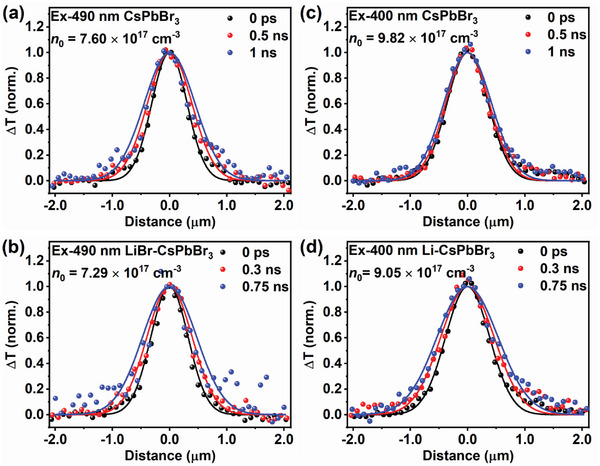
1D TAM images fitted by Gaussian function at different delay times, with the maximum Δ*T* signal normalized. a,b) CsPbBr_3_ and LiBr‐CsPbBr_3_ films under 490 nm excitation, *n*_0_ ≈7.45×10^17^ cm^−3^. c,d) CsPbBr_3_ and LiBr‐CsPbBr_3_ films under 400 nm excitation, *n*_0_ ≈9.44×10^17^ cm^−3^.

Owing to the isotropy of carrier transport, we have used the 1D TAM imaging to measure the carrier diffusion for reducing data acquisition time. The 1D TAM images of two films with different excitation wavelengths and initial carrier densities are shown in **Figure** **3** and Figures S17,S18, S20, and S21 (Supporting Information). Similar to the 2D TAM image, we can use a Gaussian function $n\;\left(x,t\right)=\;Nexp\left[-\frac{{\left(x-x_{0}\right)}^{2}}{2\sigma {\left(t\right)}^{2}}\right]$ to get *L* ^2^ =  2*Dt*  = σ(*t*)^2^  − σ(0)^2^ and then extract the diffusion constant by linear fitting. The fitting results are shown in Figures S19 and S22 (Supporting Information) and summarized in Table S1 (Supporting Information).

It's found that the diffusion constant enlarges with the increasing initial carrier density under both of 490 nm (near band edge) and 400 nm (above the bandgap) excitations. It's possible because some carriers could be localized at some potential minima and do not participate in diffusion, while the rest carriers are delocalized and mobile.^[^ ^22^ ^]^ With the increasing of photoexcited carrier density, the relative part of delocalized carriers increases, resulting in the enlargement of the *D* value at higher pump fluence.^[^ ^22^ ^]^ However, it can't rule out the artifact caused by the bimolecular recombination of carriers when the carrier density is ≈10^18^ cm^−3^. The normalized TAM dynamics (when pump and probe beams overlapped spatially) for two samples under different initial carrier densities are shown in Figure S23 (Supporting Information). It's clear that the decay accelerates with the increase of *n*_0_. The bimolecular recombination of carriers could lead to artificial enlargement of TAM image over time which would magnify the measured diffusion constant. Therefore, we need to eliminate the effect of bimolecular recombination to get the true value of the diffusion constant.

### 2.4 Modelling the Carrier Diffusion and Recombination Dynamics

To understand the complex behavior of carrier transport in CsPbBr_3_ and LiBr‐CsPbBr_3_, it's necessary to take into account the hot carrier trapping and cooling processes, the cold carrier recombination (including monomolecular and bimolecular recombination), and the diffusion of the cold carrier. We haven't observed the diffusion of hot carriers (see the inset of Figure S16b, Supporting Information). It may be because the lifetime of the hot carrier is too short (≤ 0.3 ps, Figure S25, Supporting Information), and the hot carriers have been already trapped or cooled before they can move away. With considering all the involved processes, the following rate equations are used to describe the hot (*N_h_*), cold (*N_c_*), and trapped (*N_t_*) carriers population:^[^ ^5^, ^18^, ^23^ ^]^

(4) $$\frac{\partial N_{h}\left(x,t\right)}{\partial t}=-k_{cooling}\;N_{h}\left(x,t\right)-k_{trapping}N_{h}\left(x,t\right)$$

(5) $$\begin{array}{cc} \frac{\partial N_{c}\left(x,t\right)}{\partial t} & =D\left[\frac{\partial^{2}N_{c}\left(x,t\right)}{\partial x^{2}}\right]+k_{cooling}N_{h}\left(x,t\right)\\ & \;-AN_{c}\left(x,t\right)-BN_{c}^{2}\left(x,t\right) \end{array}$$

(6) $$\frac{\partial N_{t}\left(x,t\right)}{\partial t}=k_{trapping}\;N_{h}\left(x,t\right)-k_{trap\_decay}N_{t}\left(x,t\right)$$

where, *k_cooling_* is the cooling rate of hot carriers, and *k_trapping_* is the trapping rate of hot carriers, *D* is the diffusion constant of cold carriers, *A* and *B* are the rate constants of monomolecular and bimolecular recombination of cold carriers, *k_trap_decay_* is the decay rate of trapped carriers. The Auger recombination is ignored under our experimental condition (*n*_0_ = 10^17^–10^18^ cm^−3^).^[^ ^24^ ^]^ There is no hot carrier present under near‐band edge excitation (490 nm). So the equations could be simplified as:

(7) $$\frac{\partial N_{c}\left(x,t\right)}{\partial t}=\;D\left[\frac{\partial^{2}N_{c}\left(x,t\right)}{\partial x^{2}}\right]-AN_{c}\left(x,t\right)-BN_{c}^{2}\left(x,t\right)$$

Note that the monomolecular recombination is considered as trap state‐mediated usually.^[^ ^12^, ^14^ ^]^ But in this study, the trapped cold carriers could detrap easily (see TA data). So we haven't added the trapping and detrapping processes of cold carrier in our model to simplify calculations. The trap state of the cold carrier should be caused by the shallow defect.^[^ ^14^ ^]^

**Figure 4:**
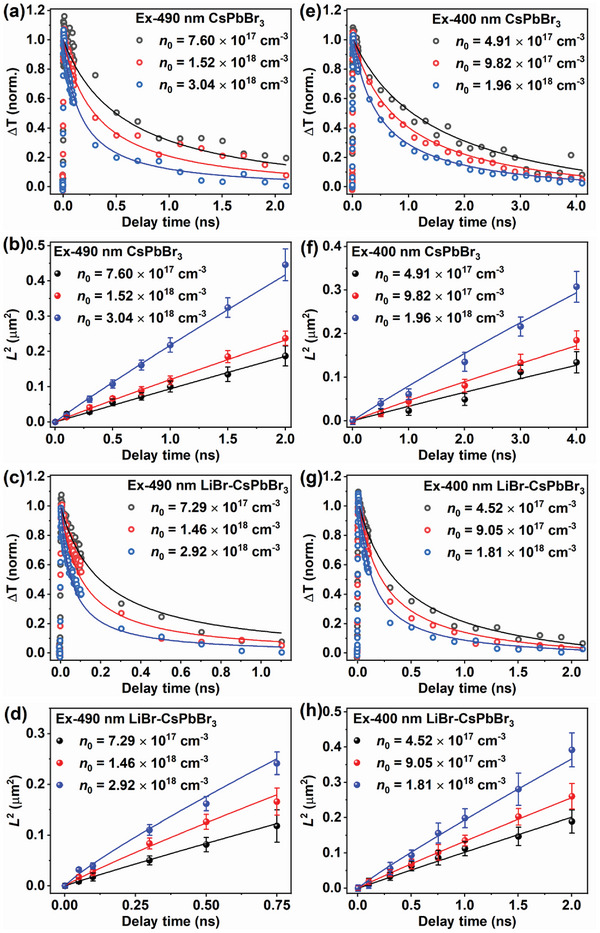
Numerical simulation of the experimental TAM dynamics and diffusion data. a,c) The normalized experimental (scatter) and simulated (solid line) kinetics of CsPbBr_3_ and LiBr‐CsPbBr_3_ with different initial carrier density *n*_0_ under 490 nm excitation. b,d) The experimental (scatter) and simulated (solid line) time evolution of *L* ^2^ for CsPbBr_3_ and LiBr‐CsPbBr_3_ with different initial carrier density *n*_0_ under 490 nm excitation. e–h) The results for 400 nm excitation. Error bars are the standard error estimated from the 1D Gaussian fitting to the spatial intensity distribution.

The Equations (4),(5),(6), and (7) were used to simulate the experimental TAM dynamics and diffusion data. The *D* value was varied to best fit the time evolution of the spatial profile of the cold carriers. The *A* value was obtained by fitting the TRPL data of CsPbBr_3_ and LiBr‐CsPbBr_3_ under very low pump fluence (see Figure S24, Supporting Information and accompanying discussion for the details). The *B* value was varied to best fit the power‐dependent dynamics of the cold carriers under 490 nm excitation. The hot carrier cooling time constant was determined by fitting the dropping of the carrier temperature, as shown in Figure S25 (Supporting Information). The *k_trapping_* value was varied to best fit the power‐dependent dynamics of the cold carriers under 400 nm excitation. Note that the decay of carriers are much slower under 400 nm excitation than that under 490 nm excitation (Figure S23, Supporting Information), but the *A* and *B* values should be independent on excitation wavelength. It's because the hot carrier trapping has reduced the density of cold carriers under 400 nm excitation. So the dynamics of the cold carriers under 400 nm excitation can help to obtain *k_trapping_*. The trapped hot carriers are hard to detrap, so the trap state of the hot carrier should be caused by the deep defect.^[^ ^14^ ^]^ We think that the trapped hot carriers haven't participated in the recombination and diffusion processes of cold carriers in the period that we measured. So the lifetime of the trapped carriers (*k_trap_decay_*) doesn't affect the simulation. The simulation results are shown in **Figure** **4**. The entire experimental dataset is reproduced satisfactorily. The rates and diffusion constants are summarized in **Table** **1**.

**Table 1:** Fitting parameters obtained from the simulation of TAM data of CsPbBr_3_ and LiBr‐CsPbBr_3_ films.

sample	pump wavelength	*n*_0_ [cm^−3^]	*D* [cm^2^ s^−1^]	*A* [s^−1^]	*B* [cm^3^ s^−1^]	*k_cooling_* [s^−1^]	*k_trapping_* [s^−1^]
CsPbBr_3_	490 nm	7.60×10^17^	0.34	1.3×10^8^	1.2×10^−9^		
		1.52×10^18^	0.38	1.3×10^8^	1.2×10^−9^		
		3.04×10^18^	0.68	1.3×10^8^	1.2×10^−9^		
	400 nm	4.91×10^17^	0.12	1.3×10^8^	1.2×10^−9^	9.1×10^12^	4.0×10^12^
		9.82×10^17^	0.15	1.3×10^8^	1.2×10^−9^	4.8×10^12^	2.6×10^12^
		1.96×10^18^	0.25	1.3×10^8^	1.2×10^−9^	3.4×10^12^	2.0×10^12^
LiBr‐CsPbBr_3_	490 nm	7.29×10^17^	0.48	6.7×10^7^	4.1×10^−9^		
		1.46×10^18^	0.65	6.7×10^7^	4.1×10^−9^		
		2.92×10^18^	0.85	6.7×10^7^	4.1×10^−9^		
	400 nm	4.52×10^17^	0.34	6.7×10^7^	4.1×10^−9^	7.1×10^12^	2.0×10^12^
		9.05×10^17^	0.39	6.7×10^7^	4.1×10^−9^	4.3×10^12^	1.7×10^12^
		1.81×10^18^	0.52	6.7×10^7^	4.1×10^−9^	3.3×10^12^	1.4×10^12^

As shown in Table 1, The diffusion constant *D* value obtained by simulation is obviously reduced than the result of linear fitting (Table S1, Supporting Information). It is clearly that the bimolecular recombination has caused an artificial acceleration of the carrier diffusion but we can use the modeling to extract the real diffusion constant. The *D* value of LiBr‐CsPbBr_3_ is larger than CsPbBr_3_ at almost the same *n*_0_, which indicates that the LiBr passivation has improved the carrier diffusion rate.

Hot carrier cooling and trapping processes are involved for 400 nm excitation. With the increasing of initial carrier density, the cooling rate of the hot carrier slows down gradually, which is caused by the hot phonon bottleneck effect.^[^ ^16^, ^25^ ^]^ Meanwhile, the rate of hot carrier trapping slows down. Because the quantity and distribution of the defect site are intrinsic, but the traps will be all filled with carriers at higher carrier density, and then the probability of carrier trapping is reduced.^[^ ^26^ ^]^ So the increasing of initial carrier density could lead to the slowing down of the trapping rate.

It's worth noting that the simulated *D* value of 400 nm excitation (0.15 cm^2^ s^−1^) is obviously smaller than that of 490 nm excitation (0.34 cm^2^ s^−1^) for CsPbBr_3_ under similar *n*_0_ (≈8.7×10^17^ cm^−3^). The reason is that the hot carrier trapping process is competing with the cooling process. The trapped carriers can not participate in the carrier diffusion process. So the hot carrier trapping would decrease the population of cold carriers which are diffusible, then lower the carrier diffusion constant. But for LiBr‐CsPbBr_3_, this effect has been significantly weakened (0.39 cm^2^ s^−1^ for 400 nm vs 0.48 cm^2^ s^−1^ for 490 nm). It's because the hot carrier trapping rate has been slowed down by the passivation. So it's proved that LiBr can passivate the defect and lower the trapping probability of hot carriers, thus improve the carrier diffusion rate.

**Figure 5:**
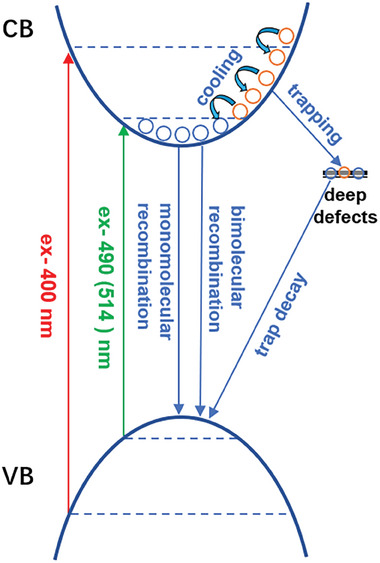
Illustration of the carrier relaxation processes of CsPbBr_3_ and LiBr‐CsPbBr_3_ films. Note that only the dynamic processes of the electron are drawn up, but the dynamic processes of the hole are not shown for clarity.

The carrier relaxation processes of CsPbBr_3_ and LiBr‐CsPbBr_3_ films could be illustrated as in **Figure** **5**. With near band edge excitation (490/514 nm), the excited cold carriers would recombine via monomolecular and bimolecular recombination processes. With above bandgap excitation (400 nm), the hot carrier cooling and trapping processes would occur simultaneously. The hot carrier trapping may lead to the reducing of the total population of cold carriers. The cold carrier would recombine later like the situation of near‐band edge excitation.

## 3 Conclusion

In summary, we have studied the carrier dynamics and carrier diffusion processes of CsPbBr_3_ and LiBr‐CsPbBr_3_ perovskite films by the means of TA and TAM. It's found that there is a fast hot carrier trapping process with the above bandgap excitation, and the hot carrier trapping would decrease the population of cold carriers which are diffusible, then lower the carrier diffusion constant. It's proved that LiBr can passivate the defect and lower the trapping probability of hot carriers, thus improve the carrier diffusion rate. Our finding demonstrates the influence of hot carrier trapping to the carrier diffusion, and provides a new insight to the carrier dynamics processes in all‐inorganic CsPbBr_3_ perovskite film.

## 4 Experimental Section

### Materials

Lead (II) bromide (99.999%, metal basis), cesium bromide (99.999%, metal basis), and lithium bromide (99.999%, metal basis) were purchased from Alfa Aesar. DMSO (Dimethyl sulfoxide, 99.7%, extra dry), and DMF (Dimethylformamide, 99.9%) were purchased from J&K. 18‐Crown‐6 (99%) were purchased from Acros. All chemicals were used without further purification.

### Sample Preparation

The two polycrystalline perovskite films were synthesized following the method previously reported by Wu et al.^[^ ^8^ ^]^ For CsPbBr_3_ precursor: CsBr (0.22 m), PbBr_2_ (0.2 m) and 18‐Crown‐6 (3.5 mg mL^−1^) were dissolved in DMSO and DMF (4:1 volume ratio), then stirring for 2 h (at 60 °C). For LiBr‐CsPbBr_3_ precursor: LiBr (0.04 m, it accounts for 20% of PbBr_2_ which is the maximum concentration for passivating in the literature)^[^ ^8^ ^]^ was added into the pristine solution before stirring. Both precursor solutions were filtered by a polytetrafluoroethylene filter (0.45 µm) before using. A two‐step process of 1000 r.p.m. for 5 s and 3000 r.p.m for 55 s was applied to spin coat the perovskite films. Finally, the films were annealed at 100 °C for 5 min. The films were prepared on a 1 mm thick glass substrate and encapsulated by a 0.17 mm thick coverslip to rule out the effects from air.

### Steady‐State Absorption Spectroscopy and Photoluminescence Microscopy

The UV–vis absorption spectra were recorded by using a U‐3900 spectrometer (Hitachi). The PL microscopy images were gotten by a fluorescence microscope (E200‐F with a fluorescence attachment, Nikon).

### Scanning Electron Microscope and X‐Ray Diffraction

SEM images were obtained by a SU‐8010 (Hitachi, Japan) scanning electron microscope. The prepared perovskite film was truncated and the cross‐section was taken to determine the thickness of the film. XRD measurements were performed with Bruker D8 X‐ray diffractometer (Bruker Corporation, Germany).

### Photoluminescence (PL) Spectra and Time‐Resolved Photoluminescence (TRPL) Measurements

A home‐made system was used for the PL spectra and TRPL measurements. Output from a picosecond pulse laser of 453 nm (PDL 800‐D, Picoquant) was used as the excitation light, which was focused on the perovskite film by a lens (f = 50 mm). The PL spectra were recorded by a spectrometer (Kymera 193i, Andor) with an EMCCD camera (Newton EMCCD, Andor). TRPL signal was acquired by a single‐photon avalanche diode (PDM, PicoQuant) and a TCSPC module (PicoHarp 300, PicoQuant).

### Transient Absorption Spectroscopy

The femtosecond transient absorption (TA) spectra were measured by an ultrafast transient absorption spectrometer (Harpia‐TA, Light Conversion). A pulse with 800 nm generated by the amplified femtosecond Ti: sapphire laser (40 fs, 1 kHz, Coherent Astrella) was split into two beams. One beam went through an optical parametric amplifier (TOPAS‐C, Coherent) to generate the pump beam. The other beam was focused on a 2 mm thick CaF_2_ plate to produce continuum white light as the probe beam.

### Transient Absorption Microscopy (TAM)

The TAM images of perovskite films were taken by a home‐built TAM system that has been described in the previous publication.^[^ ^19^ ^]^ Briefly, the two output beams from an ORPHEUS‐Twins OPA (Light Conversion) pumped by an amplified femtosecond laser (1030 nm, 600 kHz, PHAROS, Light Conversion) served as the pump and probe, respectively. An acousto‐optic modulator (Gooch and Housego) was used to modulate the pump beam. The mechanical delay stage (DDS600‐E, Thorlabs) was used to delay the probe with respect to the pump. For the diffusion images, a 2D galvo mirror (GVS012, Thorlabs) was used to scan the probe beam spatially relative to the pump beam. The pump and probe were collinearly focused on the film by using an objective (CFI Plan Apo, 60×, NA = 0.95, Nikon). A 500 nm long pass filter was used to reject the pump light. The transmitted probe beam was detected by an avalanche photodiode (Hamamatsu). The pump‐induced change in the probe transmission (Δ*T*) was extracted by a lock‐in amplifier. To achieve pump–probe synchronous scanning, the optical path of the pump light was changed, so that the pump light and the probe light were collinear first and then passed through the 2D galvo mirror. By measuring the transmittance of the pump beam when scanned on an Ag nanowire (width = 90 nm), the obtained focused pump spot size was ≈418 nm for the 490 nm pump and 410 nm for the 400 nm pump.

## Conflict of Interest

The authors declare no conflict of interest.

## Supporting information

Supporting Information

## Data Availability

The data that support the findings of this study are available from the corresponding author upon reasonable request.
