# Supplementary material for: Hot Carrier Trapping and It's Influence to the Carrier Diffusion in CsPbBr3 Perovskite Film Revealed by Transient Absorption Microscopy
Source: Adv Sci (Weinh). 2024 May 10;11(28):2403507. doi: 10.1002/advs.202403507 (PMC11267283; doi:10.1002/advs.202403507)
Supplement: Supplementary file 1 — Supporting Information [file ADVS-11-2403507-s001.pdf]

ADVANCED SCIENCE
Open Access

Supporting Information

for *Adv. Sci.*, DOI 10.1002/advs.202403507

Hot Carrier Trapping and It's Influence to the Carrier Diffusion in $CsPbBr_3$ Perovskite Film Revealed by Transient Absorption Microscopy

*Jianchang Lv, Ao Liu, Danli Shi, Minjie Li, Xi Liu and Yan Wan**

Supporting Information for

Hot Carrier Trapping and It's Influence to the Carrier Diffusion in $CsPbBr_3$ Perovskite Film Revealed by Transient Absorption Microscopy

*Jianchang Lv,[1] Ao Liu,[1] Danli Shi,[1] Minjie Li,[1] Xi Liu,[1] Yan Wan[1]**

[1]College of Chemistry, Beijing Normal University, Beijing, 100875, P. R. China.

Corresponding author

*Yan Wan. E-mail: wanyan@bnu.edu.cn

Supplementary Note

Calculation of the initial carrier density.

The absorption coefficient (α) is estimated by:[1]

$$\alpha = \frac{A}{\log_{10} e \cdot d} = \frac{A}{0.434d} \quad \text{(S1)}$$

, where *d* is the thickness of perovskite film and *A* is the absorbance.

The initial carrier density (n_0) is calculated by:

$$n_0 = \frac{F\alpha}{h\omega} \quad \text{(S2)}$$

Where *F* is the pump fluence (determined by pump energy / pump facula area), $h\omega$ is the photon energy.

In addition, the critical carrier density can be calculated by:[2]

$$n_{crit} = \frac{k_B T / E_B}{11\pi\alpha_X^3} \quad \text{(S3)}$$

, where E_B is the exciton binding energy, α_x is the exciton Bohr radius. E_B is 40 meV and α_x is 7 nm for $CsPbBr_3$.[3] So n_{crit} is 5.5×10^{16} cm^{-3}. In both our TA experiment and TAM experiment, the minimum initial carrier density exceeded this value, so the effect of excitons should be ignored.

Supplementary Figures and Tables

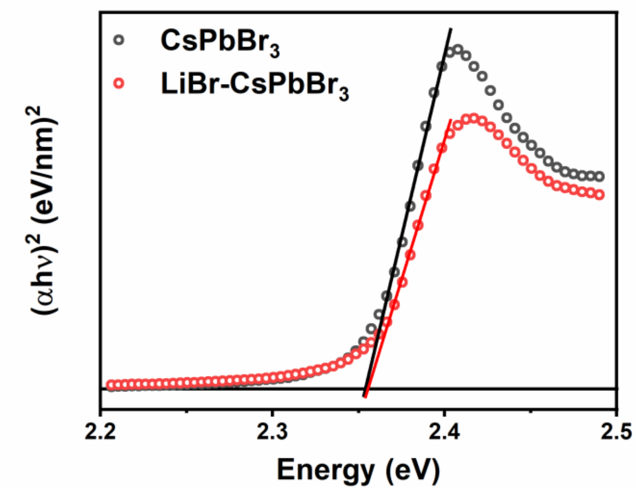

Figure S1. Tauc plots[4] showing the dependence of $(\alpha h\nu)^2$ of perovskite films upon the incident photon energy ($h\nu$).

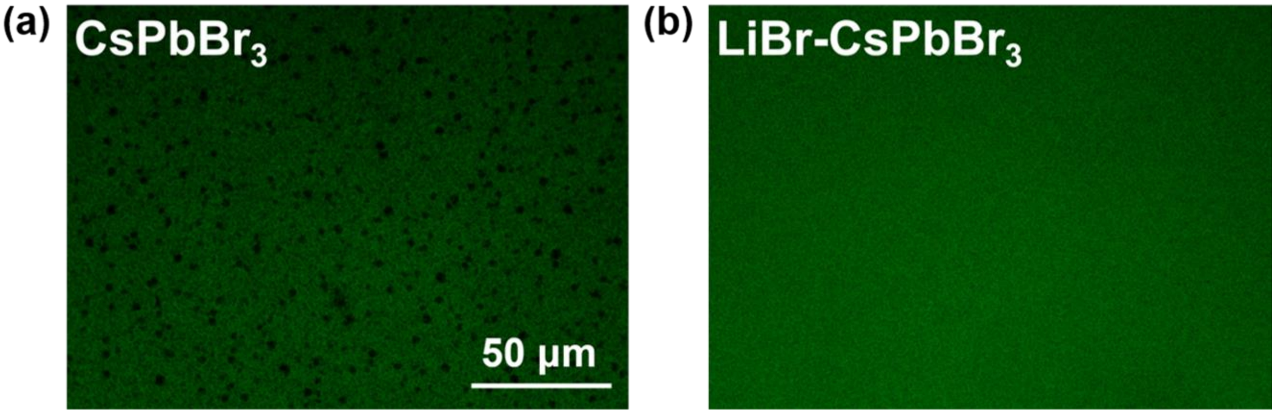

Figure S2. The PL images of (a) $CsPbBr_3$ and (b) LiBr-$CsPbBr_3$ films obtained by the PL microscope (~450 nm excitation).

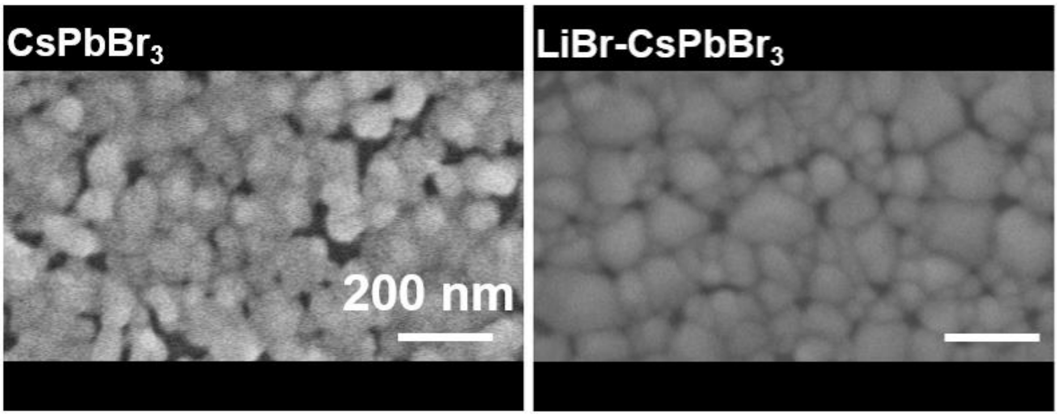

Figure S3. The scanning electron microscope (SEM) images of the surface of $CsPbBr_3$ and LiBr-$CsPbBr_3$ films.

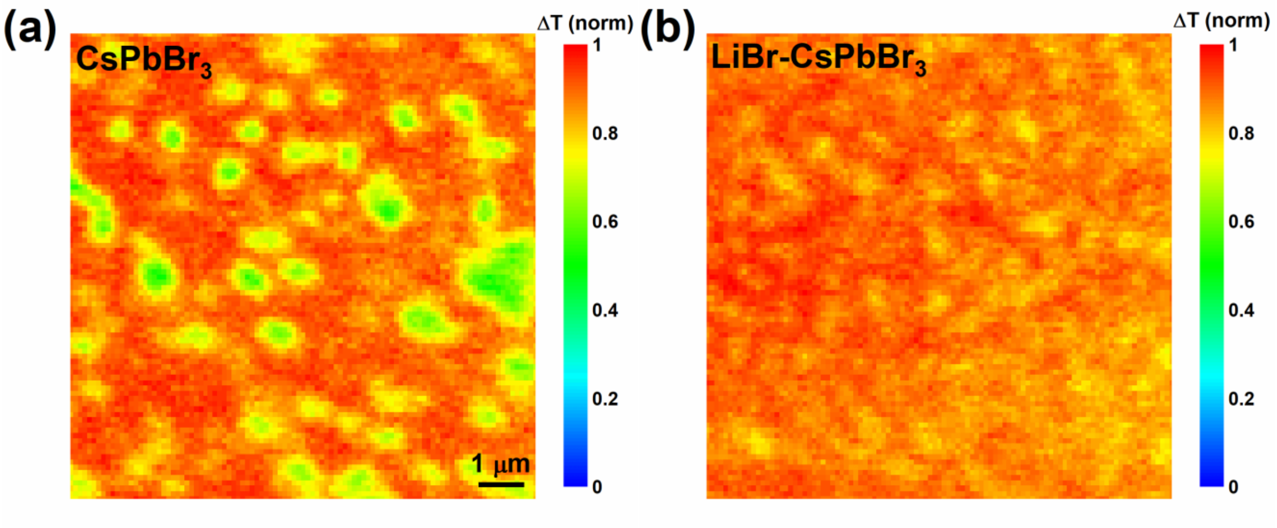

Figure S4. The TAM synchronous scanning images of (a) $CsPbBr_3$ and (b) LiBr-$CsPbBr_3$ films. Pump and probe beams are overlapped in space (pump at 400 nm, probe at 520 nm). The pump probe delay is at 0 ps.

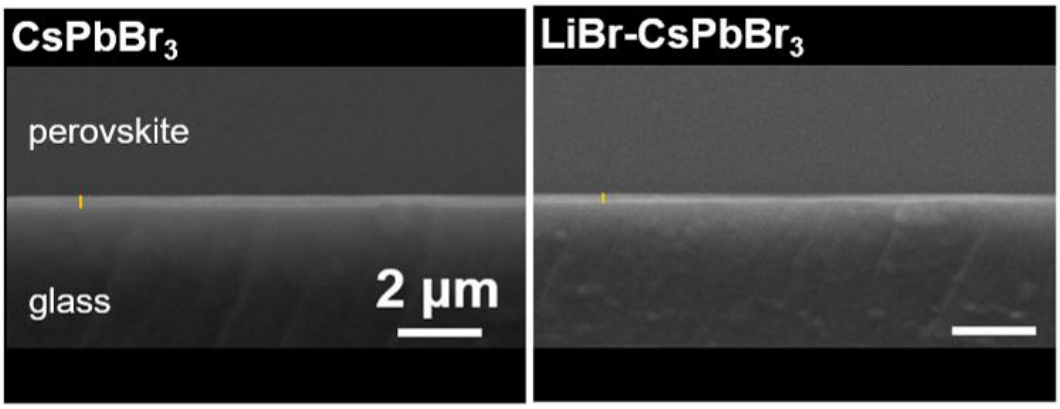

Figure S5. The scanning electron microscope (SEM) images of the cross-section of $CsPbBr_3$ and LiBr-$CsPbBr_3$ films.

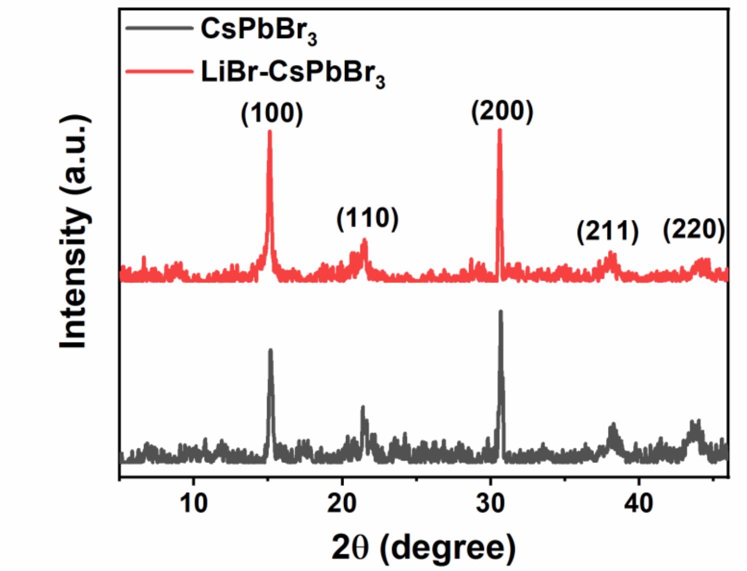

Figure S6. XRD spectra of $CsPbBr_3$ and LiBr-$CsPbBr_3$ films.

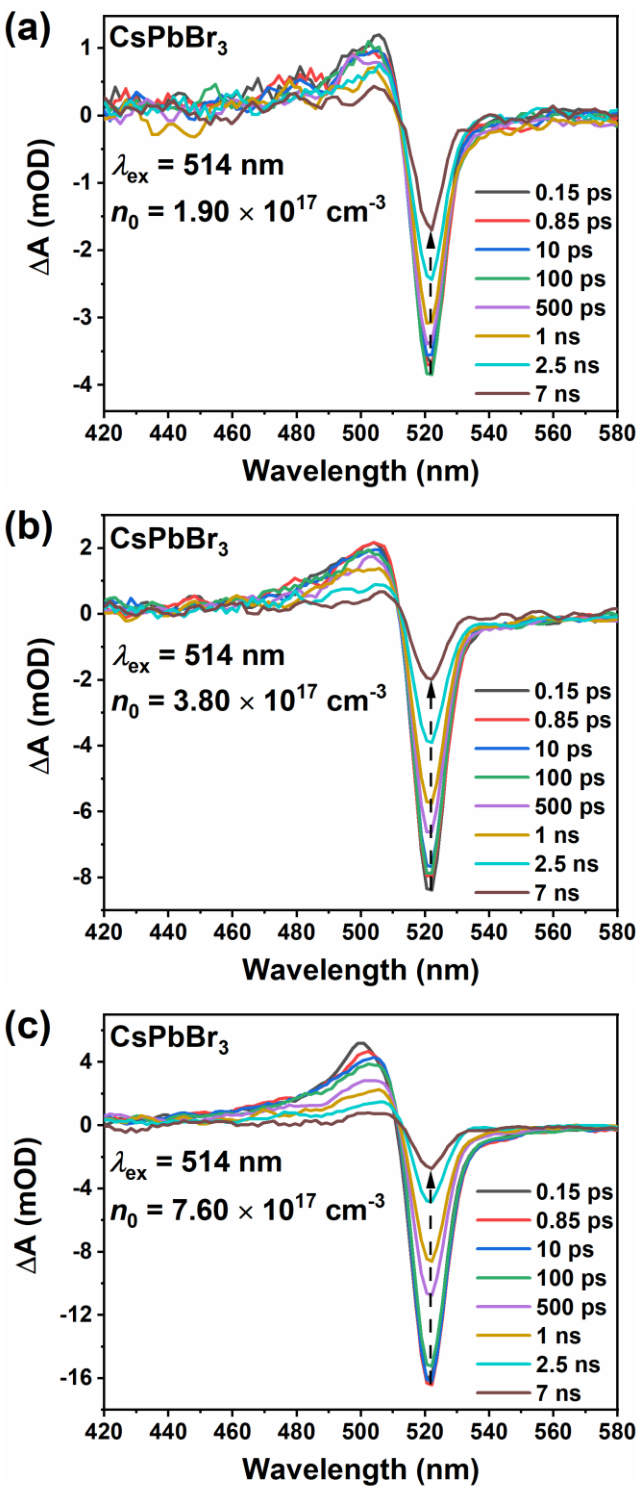

Figure S7. The TA spectra of $CsPbBr_3$ at several delay times under 514 nm excitation with different initial carrier density (n_0).

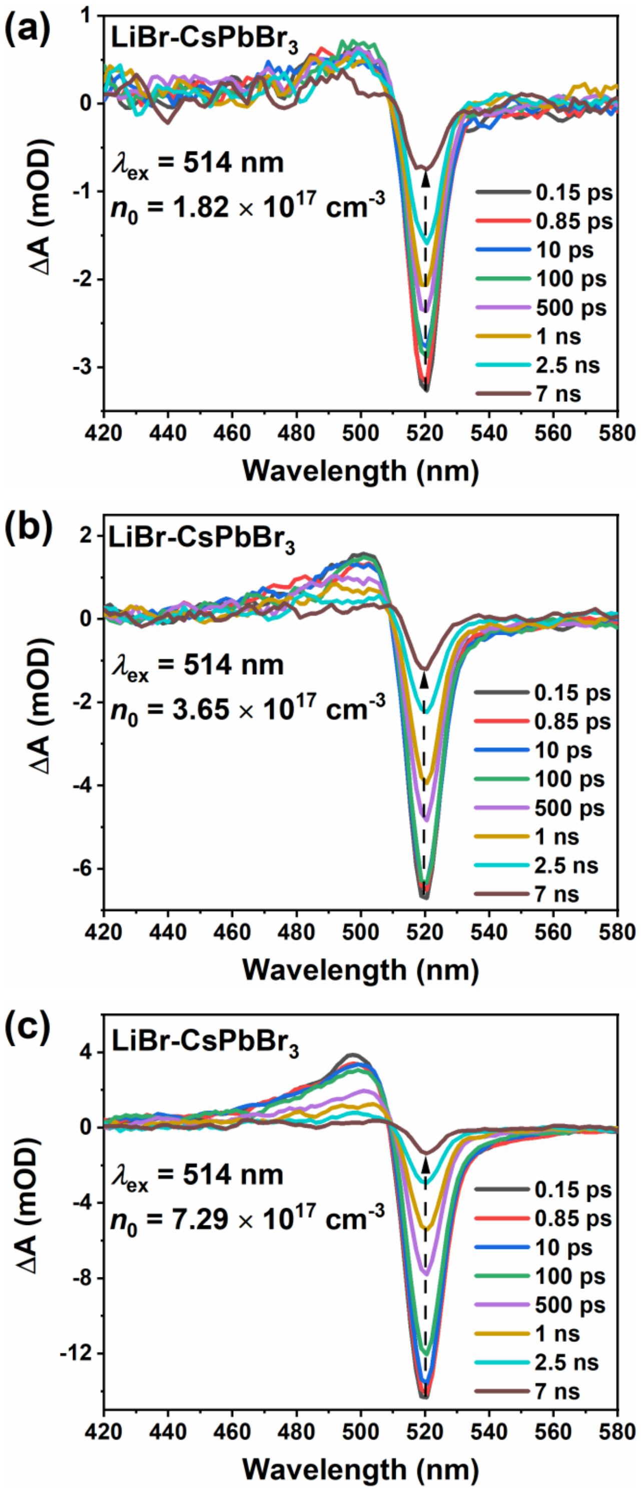

Figure S8. The TA spectra of LiBr-$CsPbBr_3$ at several delay times under 514 nm excitation with different initial carrier density (n_0).

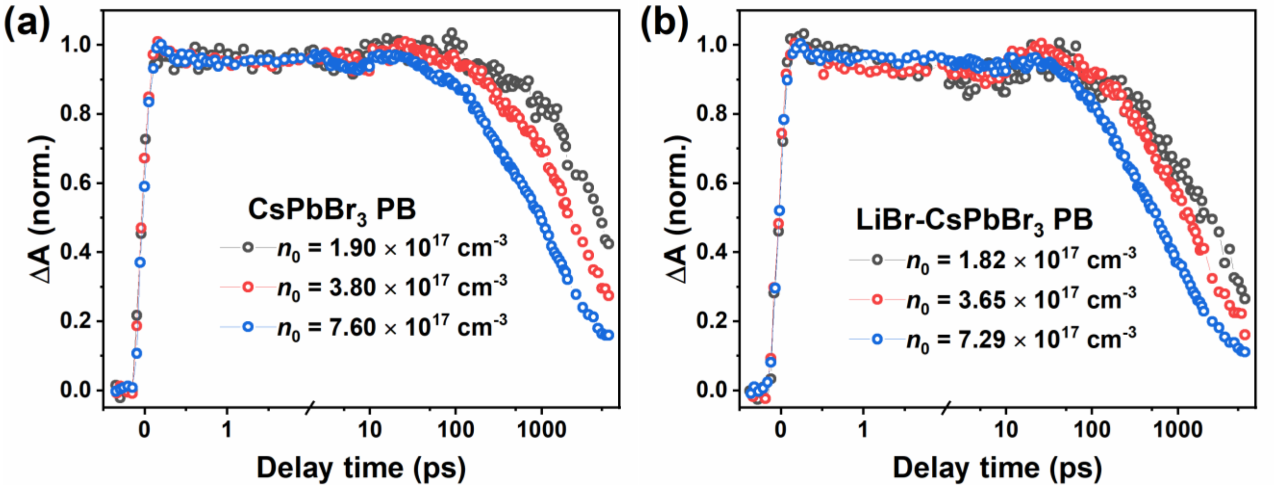

Figure S9. Normalized PB kinetics of (a) $CsPbBr_3$ and (b) LiBr-$CsPbBr_3$ with different initial carrier density. Pumped at 514 nm, probed at 520 nm.

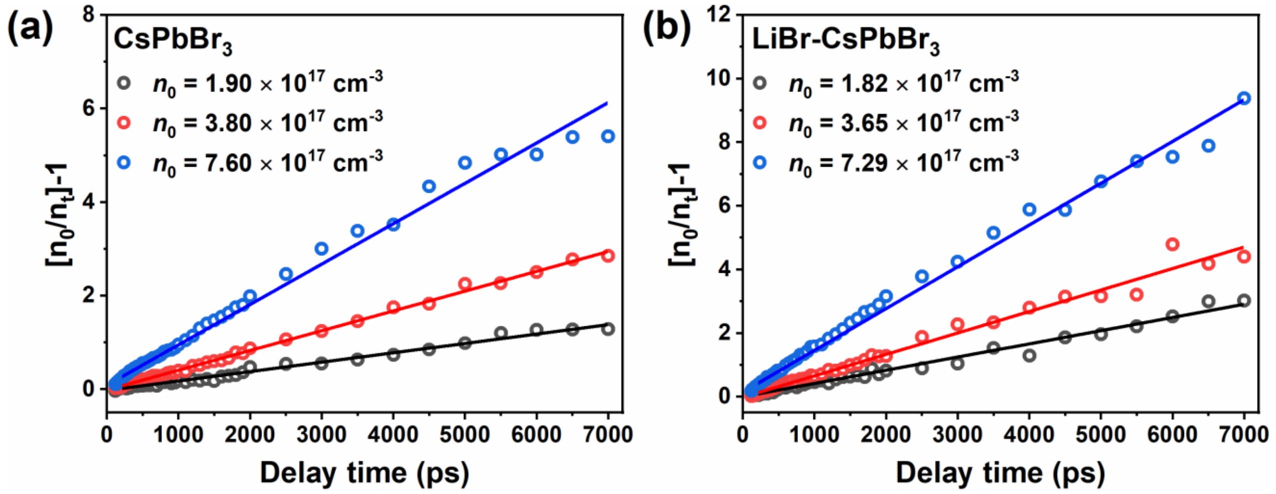

Figure S10. Linear fits to the bimolecular recombination rate equation $n_0/n_t - 1 = Bn_0t$ for (a) $CsPbBr_3$ and (b) LiBr-$CsPbBr_3$ with different initial carrier density under 514 nm excitation (probed at 520 nm). The slope is equal to Bn_0, where n_0 is the initial photogenerated carrier density and B is the rate constant for the bimolecular recombination.

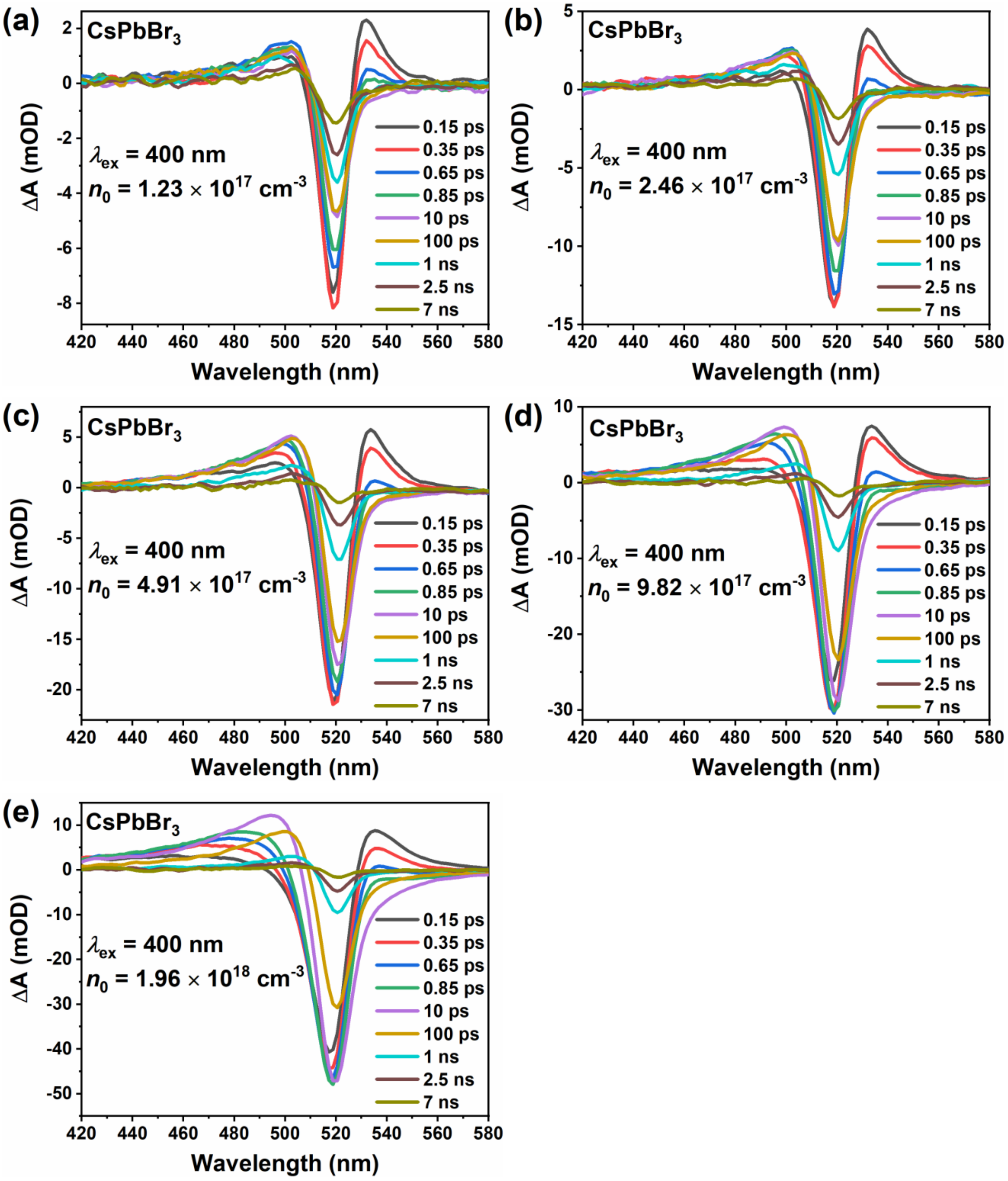

Figure S11. The TA spectra of $CsPbBr_3$ at several delay times under 400 nm excitation with different initial carrier density (n_0).

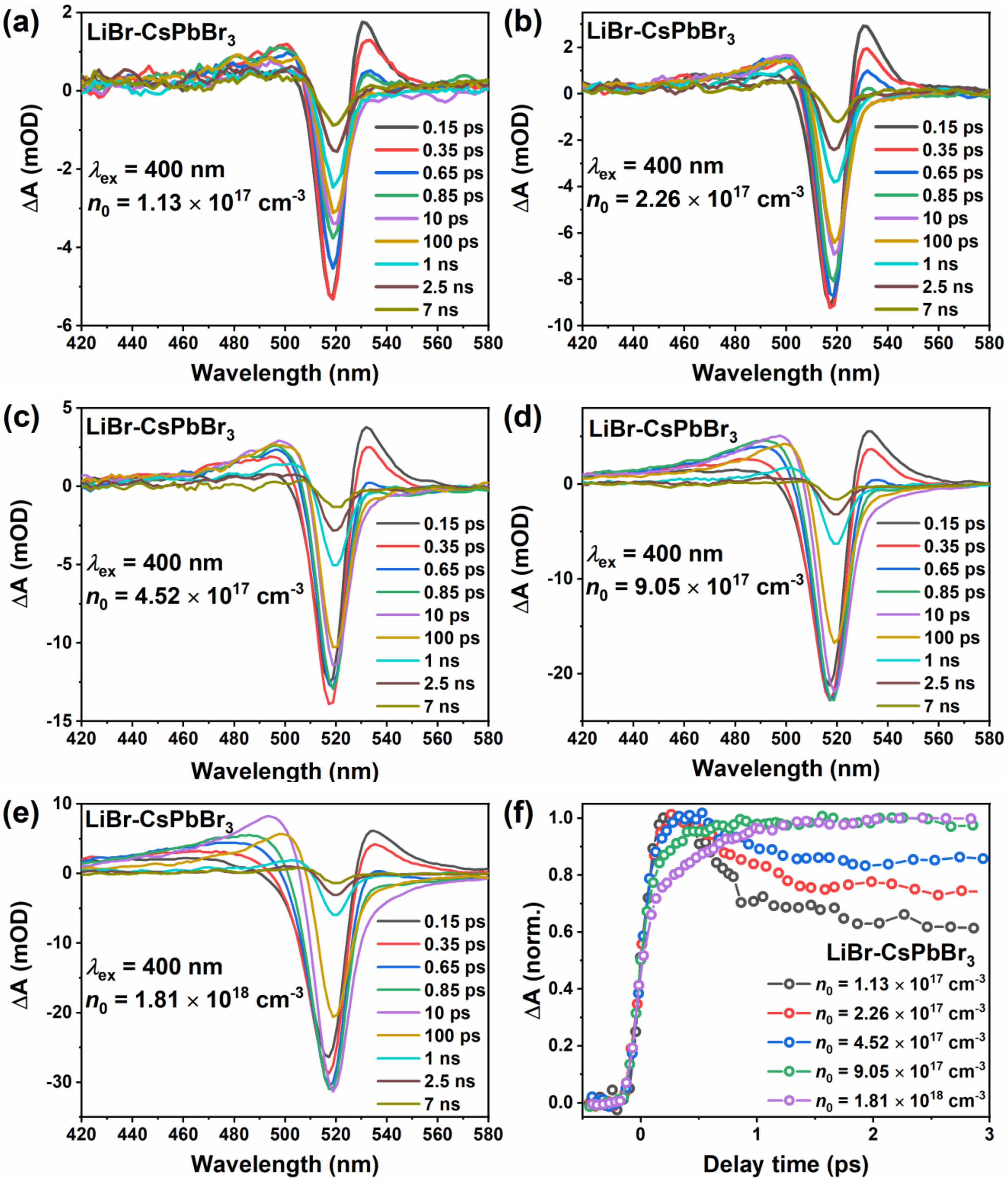

Figure S12. (a-e) The TA spectra of LiBr-$CsPbBr_3$ at several delay times under 400 nm excitation with different initial carrier density (n_0). (f) Normalized kinetic profiles probed at 520 nm in the early time with different initial carrier density for LiBr-$CsPbBr_3$ film under 400 nm excitation.

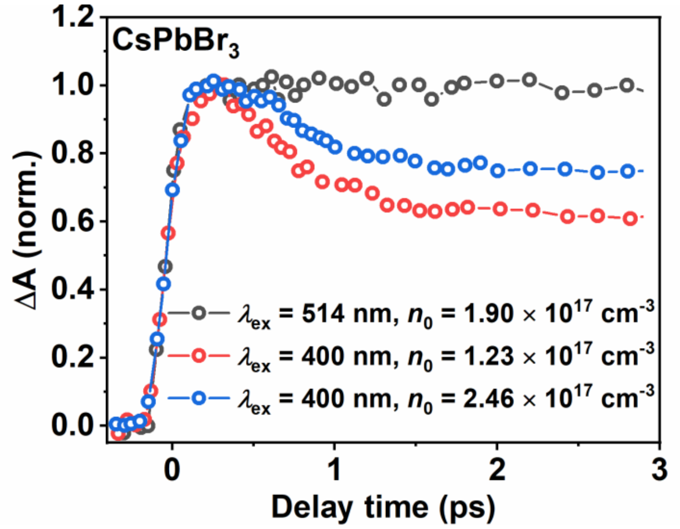

Figure S13. Normalized early time PB kinetics of $CsPbBr_3$ with different excitation wavelength at lower initial carrier density. Probed at 520 nm.

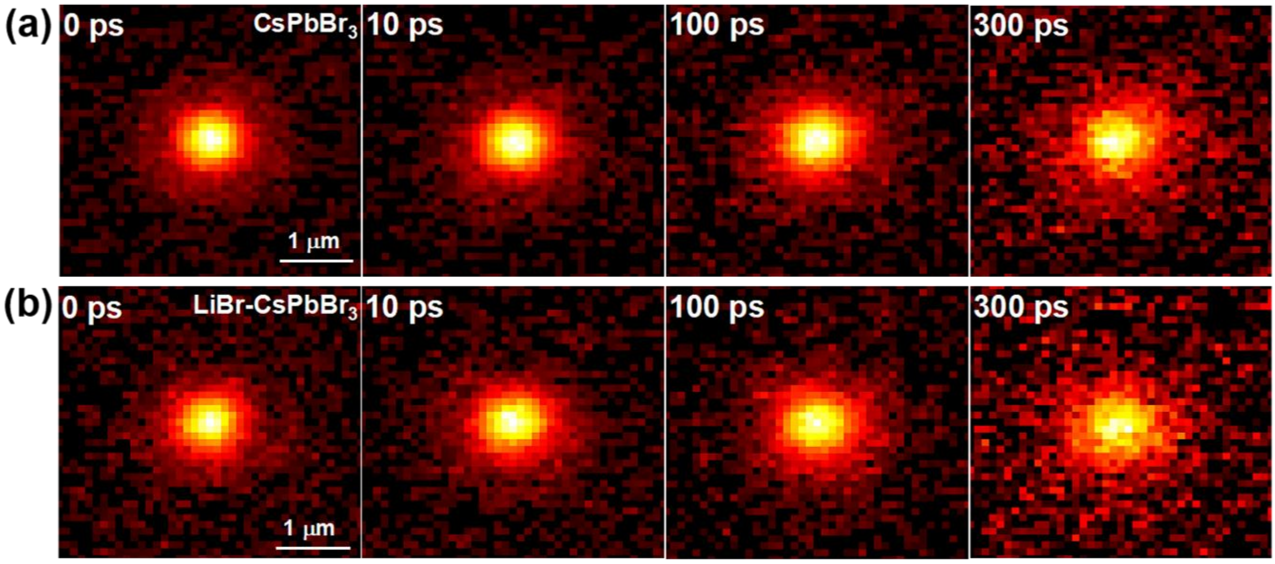

Figure S14. TAM images of (a) $CsPbBr_3$ and (b) LiBr-$CsPbBr_3$ pumped at 490 nm (n_0 ~3.0×10^{18} cm^{-3}) and probed at 520 nm. The color scale represents the intensity of pump-induced differential transmission (ΔT) of the probe, and every image has been normalized by the peak value. The images show the spatial distribution of the ΔT signal measured at pump−probe delay times as labeled.

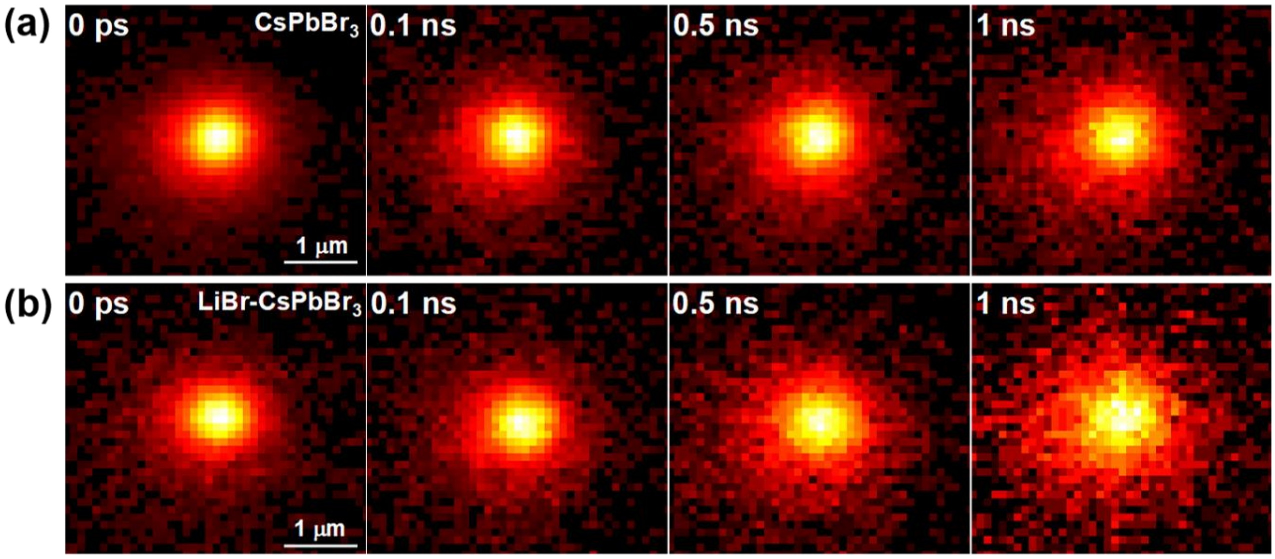

Figure S15. TAM images of (a) $CsPbBr_3$ and (b) LiBr-$CsPbBr_3$ pumped at 400 nm (n_0 ~1.9×10^{18} cm^{-3}) and probed at 520 nm. The color scale represents the intensity of pump-induced differential transmission (ΔT) of the probe, and every image has been normalized by the peak value. The images show the spatial distribution of the ΔT signal measured at pump−probe delay times as labeled.

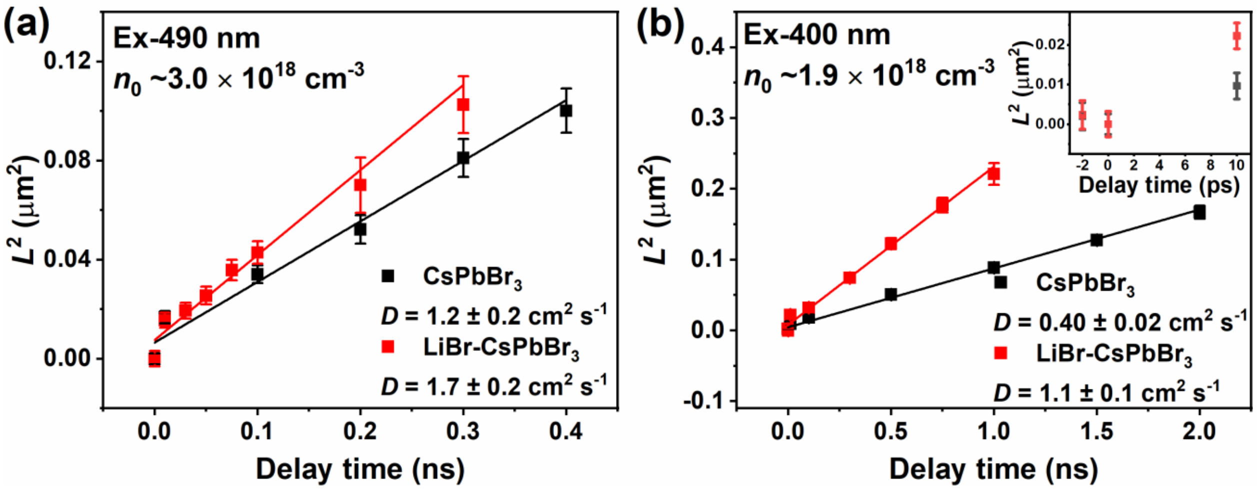

Figure S16. Time evolution of $L^2 = \sigma(t)^2 - \sigma(0)^2$ of $CsPbBr_3$ and LiBr-$CsPbBr_3$ films. Error bars are the standard error estimated from the 2D Gaussian fitting to the spatial intensity distribution. The linear fitting to eq 3 yields the diffusion constant D. The inset of (b) displays the early time evolution of L^2.

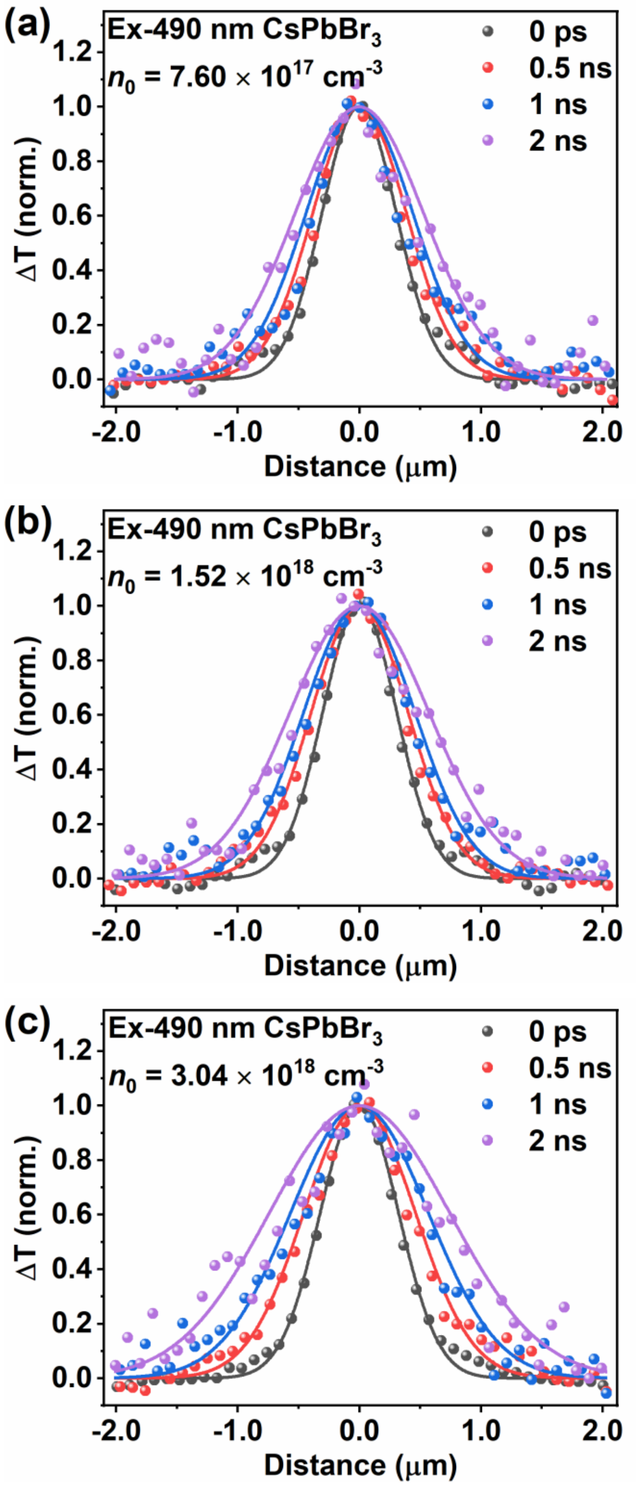

Figure S17. One-dimensional TAM images of $CsPbBr_3$ fitted by Gaussian function at different delay times, with the maximum ΔT signal normalized. Pumped at 490 nm with different initial carrier density n_0.

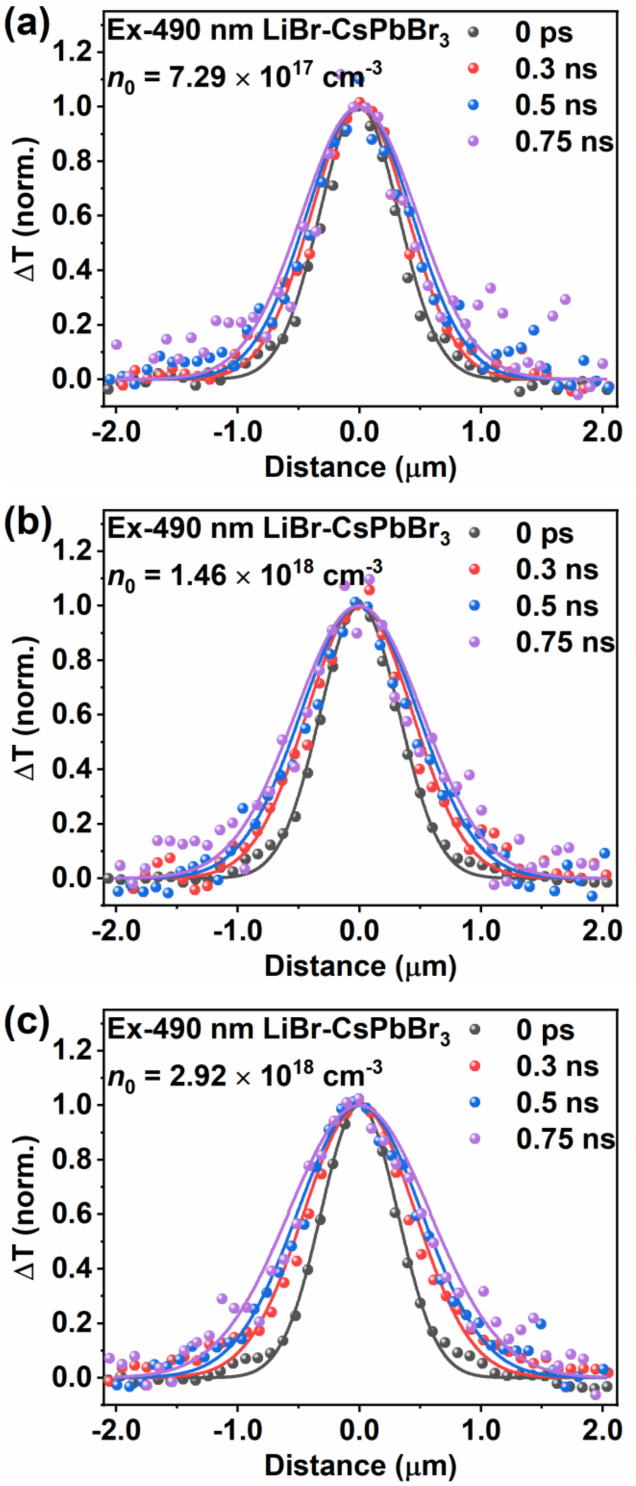

Figure S18. One-dimensional TAM images of LiBr-$CsPbBr_3$ fitted by Gaussian function at different delay times, with the maximum ΔT signal normalized. Pumped at 490 nm with different initial carrier density n_0.

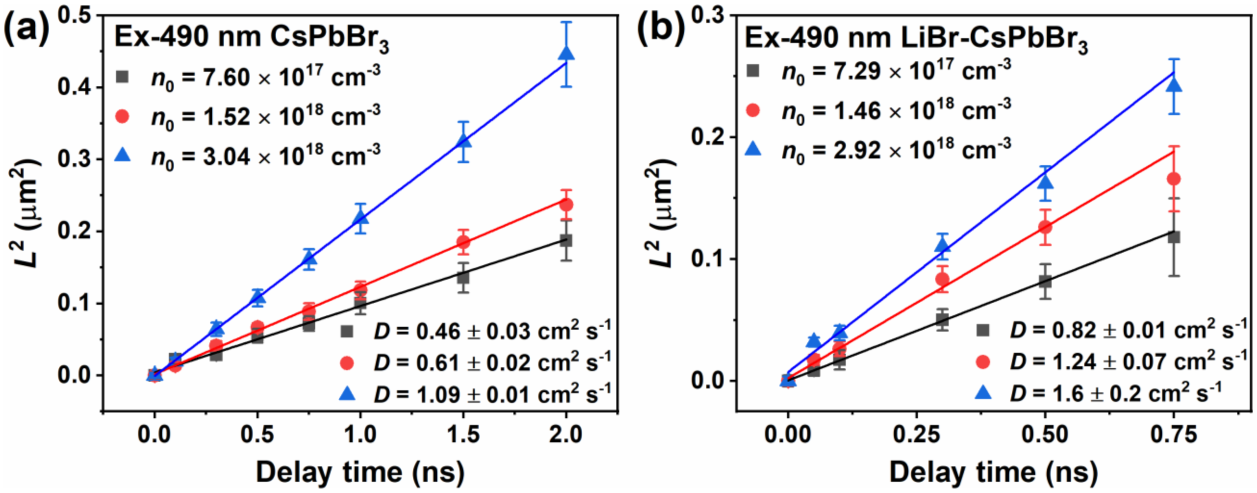

Figure S19. Time evolution of $L^2 = \sigma(t)^2 - \sigma(0)^2$ of (a) $CsPbBr_3$ and (b) LiBr-$CsPbBr_3$ films pumped at 490 nm with different initial carrier density. Error bars are the standard error estimated from the 1D Gaussian fitting to the spatial intensity distribution. The linear fitting to eq 3 yields the diffusion constant D.

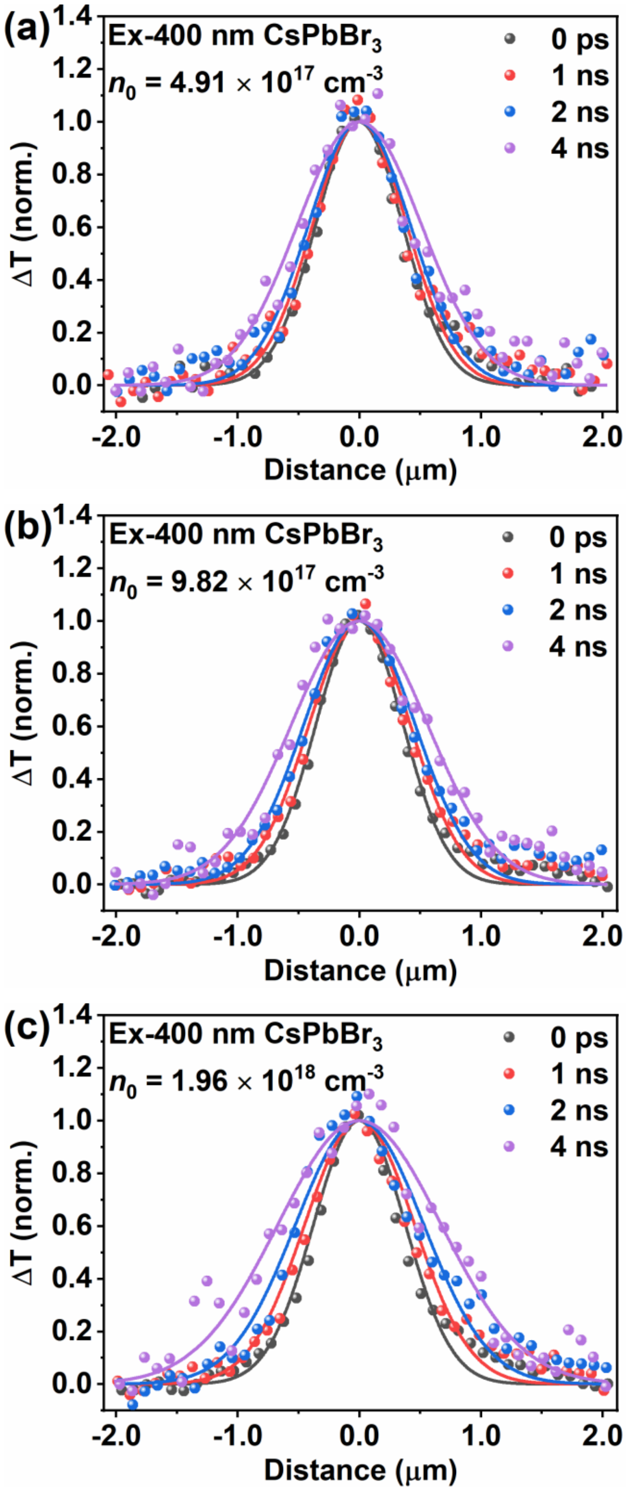

Figure S20. One-dimensional TAM images of $CsPbBr_3$ fitted by Gaussian function at different delay times, with the maximum ΔT signal normalized. Pumped at 400 nm with different initial carrier density n_0.

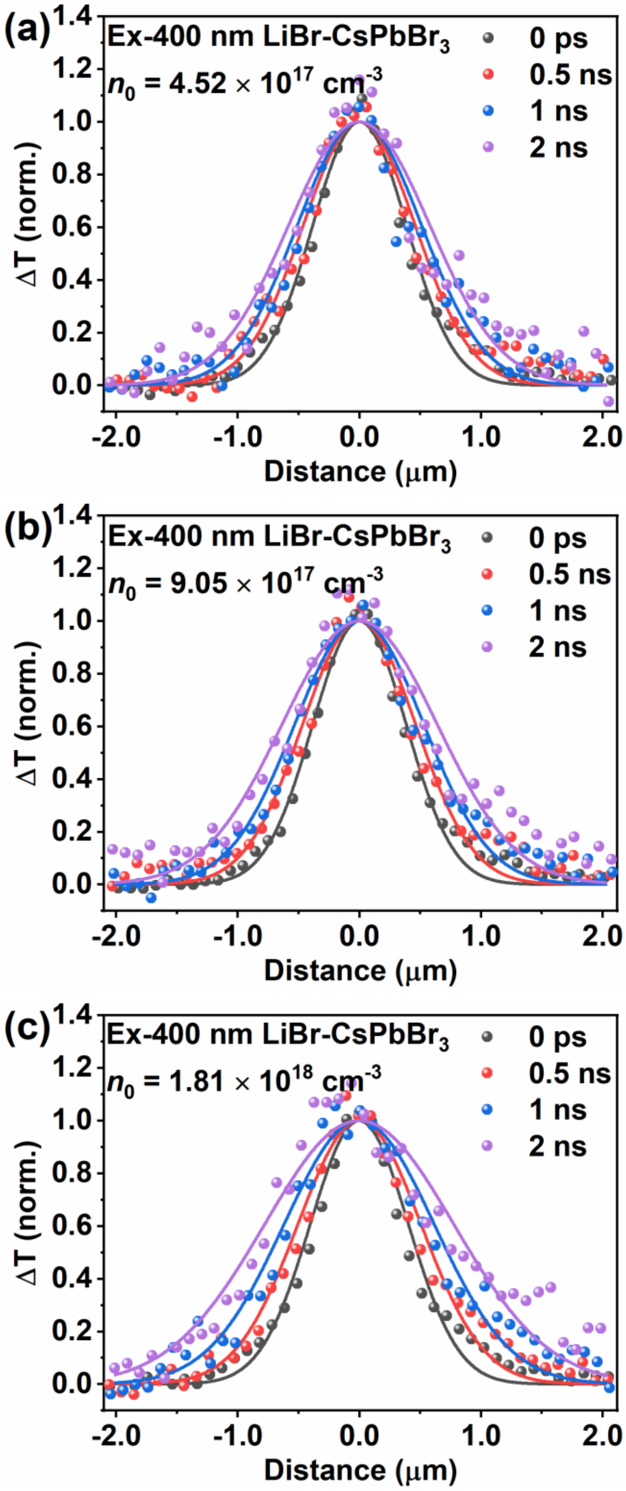

Figure S21. One-dimensional TAM images of LiBr-$CsPbBr_3$ fitted by Gaussian function at different delay times, with the maximum ΔT signal normalized. Pumped at 400 nm with different initial carrier density n_0.

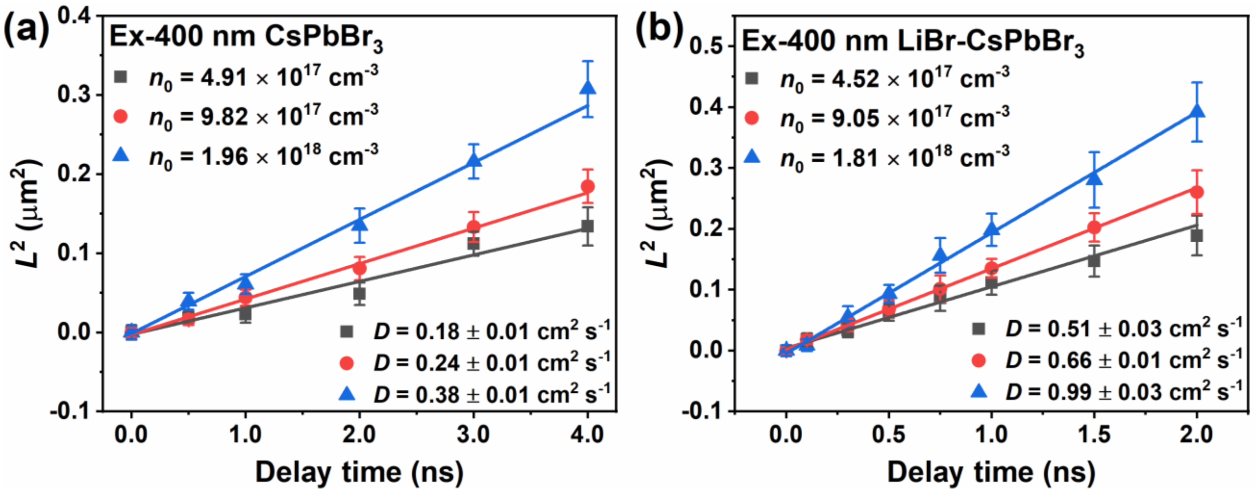

Figure S22. Time evolution of $L^2 = \sigma(t)^2 - \sigma(0)^2$ of (a) $CsPbBr_3$ and (b) LiBr-$CsPbBr_3$ films pumped at 400 nm with different initial carrier density. Error bars are the standard error estimated from the 1D Gaussian fitting to the spatial intensity distribution. The linear fitting to eq 3 yields the diffusion constant *D*.

Table S1. The fitted diffusion constant *D* of $CsPbBr_3$ and LiBr-$CsPbBr_3$ with different initial carrier density under 490 nm and 400 nm excitation.

sample	pump wavelength	n_0 (cm^{-3})	2D image *D* (cm^2 s^{-1})	1D image *D* (cm^2 s^{-1})
$CsPbBr_3$	490 nm	7.60×10^{17}		0.46 ± 0.03
		1.52×10^{18}		0.61 ± 0.02
		3.04×10^{18}	1.2 ± 0.2	1.09 ± 0.01
	400 nm	4.91×10^{17}		0.18 ± 0.01
		9.82×10^{17}		0.24 ± 0.01
		1.96×10^{18}	0.40 ± 0.02	0.38 ± 0.01
LiBr-$CsPbBr_3$	490 nm	7.29×10^{17}		0.82 ± 0.01
		1.46×10^{18}		1.24 ± 0.07
		2.92×10^{18}	1.7 ± 0.2	1.6 ± 0.2
	400 nm	4.52×10^{17}		0.51 ± 0.03
		9.05×10^{17}		0.66 ± 0.01
		1.81×10^{18}	1.1 ± 0.1	0.99 ± 0.03

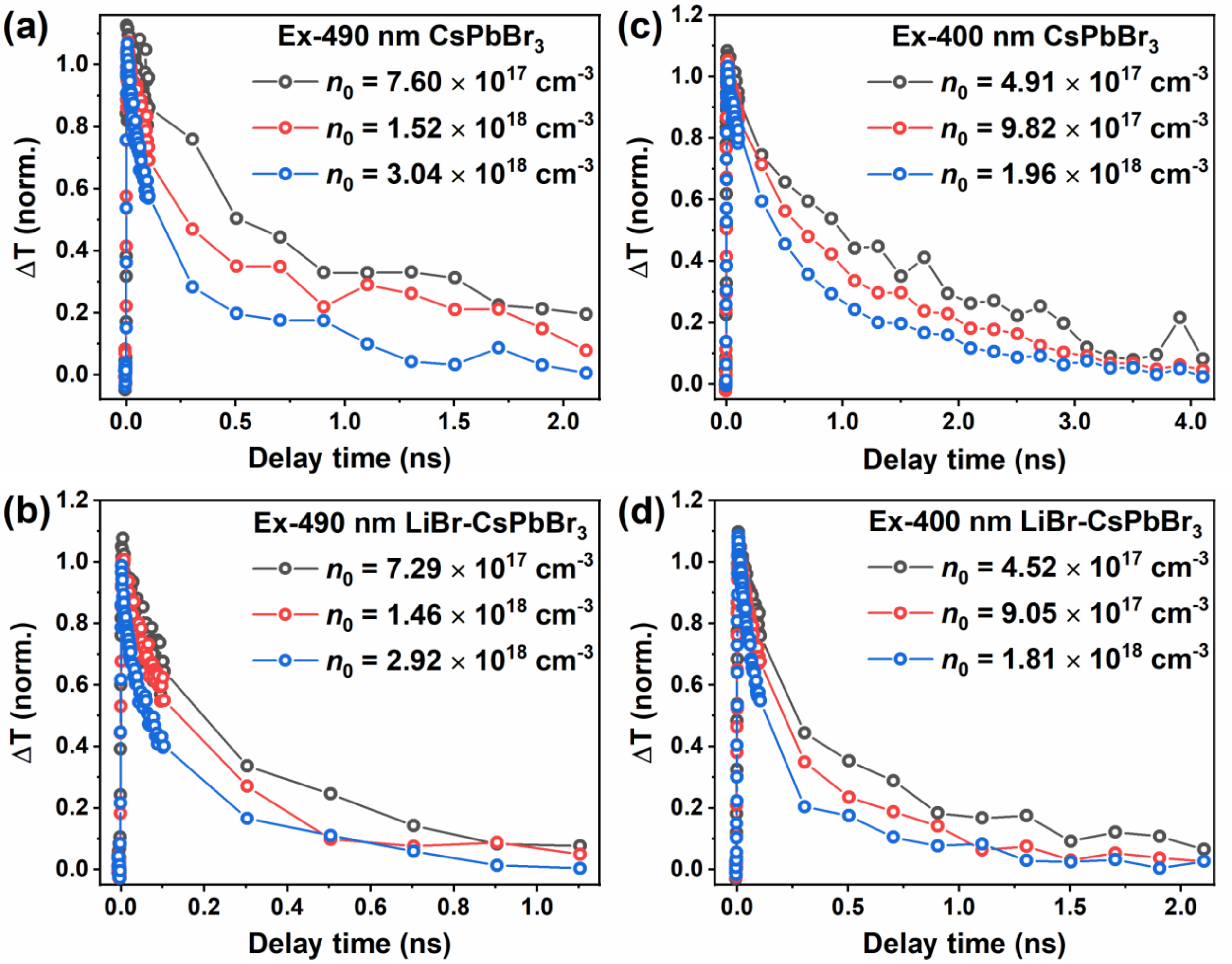

Figure S23. Normalized TAM dynamics of (a, c) $CsPbBr_3$ and (b, d) LiBr-$CsPbBr_3$ films with different initial carrier density n_0 under different excitation wavelength. The pump beam spatially overlapped with the probe beam.

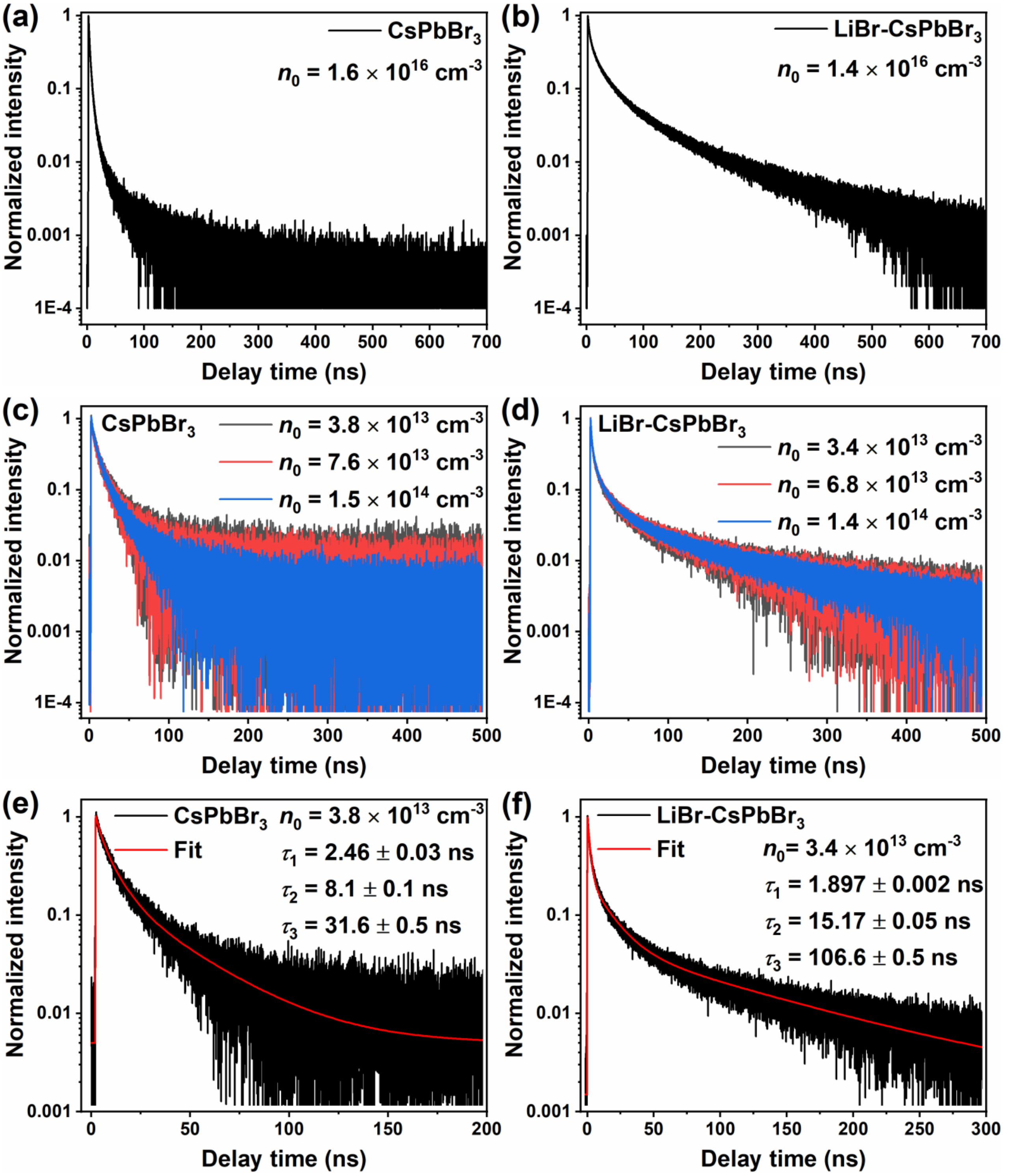

Figure S24. Time-resolved photoluminescence (TRPL) decay curves for $CsPbBr_3$ and LiBr-$CsPbBr_3$ films excited at 453 nm. (a, b) PL decay dynamics with initial carrier density n_0 ~1.5×10^{16} cm^{-3}. (c, d) PL decay dynamics without power dependence. (e, f) PL decay dynamics and exponential fitting at very low initial carrier density (n_0 ~3.6×10^{13} cm^{-3}).

We have measured the TRPL decay curves for $CsPbBr_3$ and LiBr-$CsPbBr_3$ films with initial carrier density n_0 ~1.5×10^{16} cm^{-3}. The PL lifetime of passivated perovskite film is significantly longer than

that of $CsPbBr_3$ (Figure S24a, b), agreeing with the previous report.[5] But there is still the influence of bimolecular recombination, so it's difficult to confirm the time constant of monomolecular recombination process (*A* value). Therefore, we continued reducing the pump fluence until there is no power dependence of the PL decay dynamics (n_0 ~3.6×10^{13} cm^{-3}), as shown in Figure S24c and d. The PL decay dynamics still needs three exponential fitting (see Figure S24e, f). We think that there should be three monomolecular processes: recombination of exciton (~2 ns), recombination of electron and hole that originating from the same exciton (~10 ns), and recombination of trapped carriers (30-100 ns). Note that the exciton state dominates at such a low excitation density (10^{13} cm^{-3}), but could be ignored at higher excitation density (such as in our TAM measurements). And the slowest process has little affect on the timescale of a few nanoseconds. So we have taken the rate constant of the second process (~10 ns) as the *A* value in our simulation. In addition, the reported lifetime of monomolecular recombination of $CsPbBr_3$ perovskite ranged from a few ns to tens of ns,[6] which is close to our result.

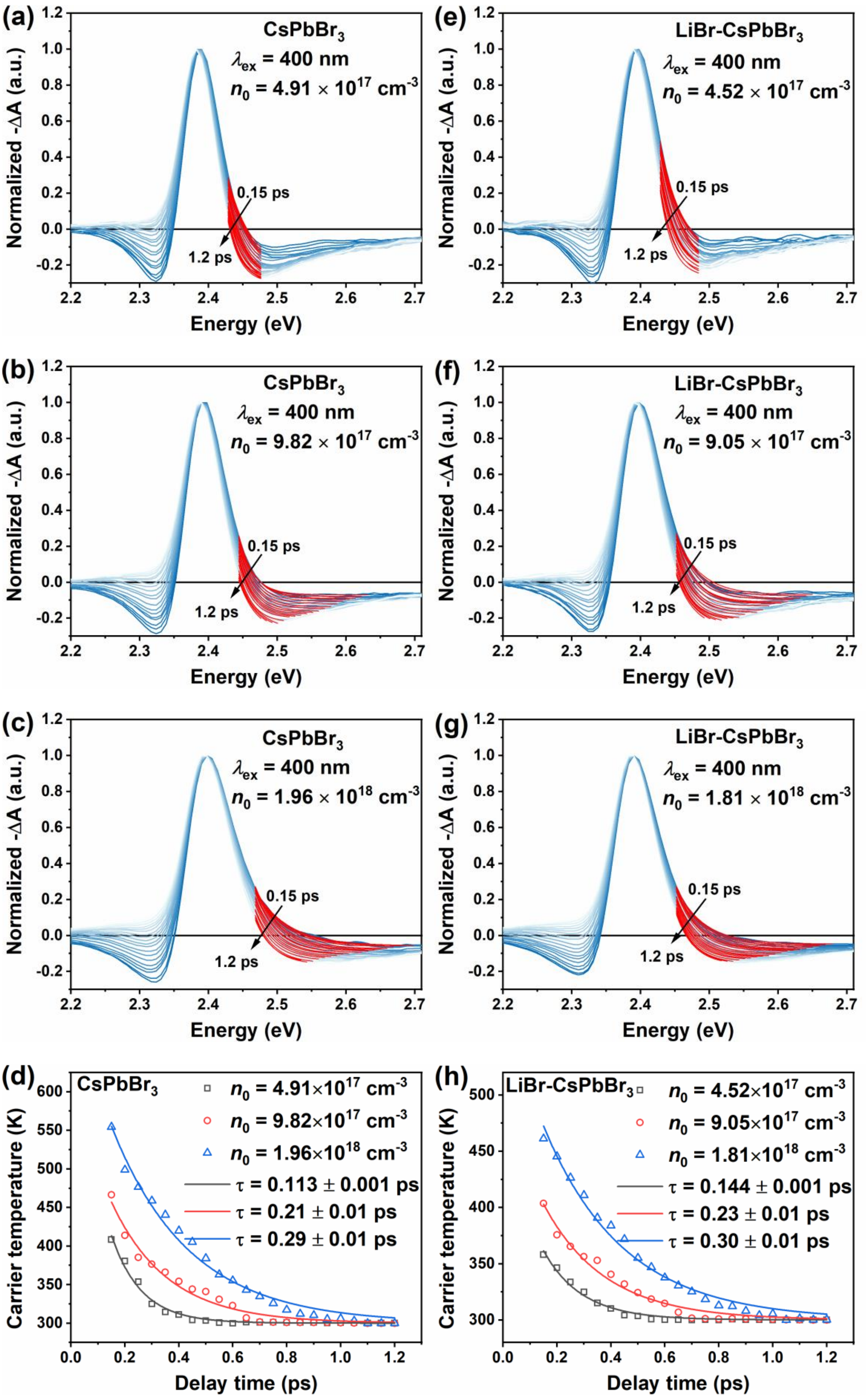
(a)
CsPbBr3
λex = 400 nm
n0 = 4.91 × 10^17 cm^-3
0.15 ps
1.2 ps
Normalized -ΔA (a.u.)
Energy (eV)
(b)
CsPbBr3
λex = 400 nm
n0 = 9.82 × 10^17 cm^-3
0.15 ps
1.2 ps
(c)
CsPbBr3
λex = 400 nm
n0 = 1.96 × 10^18 cm^-3
0.15 ps
1.2 ps
(d)
CsPbBr3
n0 = 4.91×10^17 cm^-3
n0 = 9.82×10^17 cm^-3
n0 = 1.96×10^18 cm^-3
τ = 0.113 ± 0.001 ps
τ = 0.21 ± 0.01 ps
τ = 0.29 ± 0.01 ps
Carrier temperature (K)
Delay time (ps)
(e)
LiBr-CsPbBr3
λex = 400 nm
n0 = 4.52 × 10^17 cm^-3
0.15 ps
1.2 ps
(f)
LiBr-CsPbBr3
λex = 400 nm
n0 = 9.05 × 10^17 cm^-3
0.15 ps
1.2 ps
(g)
LiBr-CsPbBr3
λex = 400 nm
n0 = 1.81 × 10^18 cm^-3
0.15 ps
1.2 ps
(h)
LiBr-CsPbBr3
n0 = 4.52×10^17 cm^-3
n0 = 9.05×10^17 cm^-3
n0 = 1.81×10^18 cm^-3
τ = 0.144 ± 0.001 ps
τ = 0.23 ± 0.01 ps
τ = 0.30 ± 0.01 ps

Figure S25. High energy tails on the normalized bleaching spectra of (a, b, c) $CsPbBr_3$ and (e, f, g) LiBr-$CsPbBr_3$ films under 400 nm excitation with different initial carrier density n_0. The blue lines are the normalized TA signals to 1 (-Δ*A*) for different time delays. The higher energy tails are well fitted by an exponential function corresponding to Maxwell-Boltzmann distribution to extract the hot carrier temperature T_c.[2,7] (d, h) The extracted carrier temperature with delay time at different carrier density. The cooling lifetimes are obtained by using a monoexponential fitting.

References

[1] M. Cesaria, A. P. Caricato, M. Martino, *J. Opt.* **2012**, *14*, 105701.

[2] X. Wang, D. Huo, X. Wang, M. Li, Y. Wang, Y. Wan, *J. Phys. Chem. Lett.* **2021**, *12*, 6907.

[3] L. Protesescu, S. Yakunin, M. I. Bodnarchuk, F. Krieg, R. Caputo, C. H. Hendon, R. X. Yang, A. Walsh, M. V. Kovalenko, *Nano Lett.* **2015**, *15*, 3692.

[4] H. Wang, X. Zhang, Q. Wu, F. Cao, D. Yang, Y. Shang, Z. Ning, W. Zhang, W. Zheng, Y. Yan, S. V. Kershaw, L. Zhang, A. L. Rogach, X. Yang, *Nat. Commun.* **2019**, *10*, 665.

[5] T. Wu, J. Li, Y. Zou, H. Xu, K. Wen, S. Wan, S. Bai, T. Song, J. A. McLeod, S. Duhm, F. Gao, B. Sun, *Angew. Chem. Int. Ed.* **2020**, *59*, 4099.

[6] a) S. Cheng, Q. Chang, Z. Wang, L. Xiao, E. E. M. Chia, H. Sun, *Adv. Opt. Mater.* **2021**, *9*, 2100564; b) J. Peng, C. Q. Xia, Y. Xu, R. Li, L. Cui, J. K. Clegg, L. M. Herz, M. B. Johnston, Q. Lin, *Nat. Commun.* **2021**, *12*, 1531; c) E. M. L. D. de Jong, G. Yamashita, L. Gomez, M. Ashida, Y. Fujiwara, T. Gregorkiewicz, *J. Phys. Chem. C* **2017**, *121*, 1941.

[7] J. Chen, M. E. Messing, K. Zheng, T. Pullerits, *J. Am. Chem. Soc.* **2019**, *141*, 3532.
